# Temporal Transcriptional Profiling of Somatic and Germ Cells Reveals Biased Lineage Priming of Sexual Fate in the Fetal Mouse Gonad

**DOI:** 10.1371/journal.pgen.1002575

**Published:** 2012-03-15

**Authors:** Samantha A. Jameson, Anirudh Natarajan, Jonah Cool, Tony DeFalco, Danielle M. Maatouk, Lindsey Mork, Steven C. Munger, Blanche Capel

**Affiliations:** Department of Cell Biology, Duke University Medical Center, Durham, North Carolina, United States of America; Stanford University School of Medicine, United States of America

## Abstract

The divergence of distinct cell populations from multipotent progenitors is poorly understood, particularly *in vivo*. The gonad is an ideal place to study this process, because it originates as a bipotential primordium where multiple distinct lineages acquire sex-specific fates as the organ differentiates as a testis or an ovary. To gain a more detailed understanding of the process of gonadal differentiation at the level of the individual cell populations, we conducted microarrays on sorted cells from XX and XY mouse gonads at three time points spanning the period when the gonadal cells transition from sexually undifferentiated progenitors to their respective sex-specific fates. We analyzed supporting cells, interstitial/stromal cells, germ cells, and endothelial cells. This work identified genes specifically depleted and enriched in each lineage as it underwent sex-specific differentiation. We determined that the sexually undifferentiated germ cell and supporting cell progenitors showed lineage priming. We found that germ cell progenitors were primed with a bias toward the male fate. In contrast, supporting cells were primed with a female bias, indicative of the robust repression program involved in the commitment to XY supporting cell fate. This study provides a molecular explanation reconciling the female default and balanced models of sex determination and represents a rich resource for the field. More importantly, it yields new insights into the mechanisms by which different cell types in a single organ adopt their respective fates.

## Author Summary

How cells diverge from a common progenitor and adopt specific fates is still poorly understood. We analyzed gene expression profiles in the distinct cell lineages of the gonad over the period when sex determination occurs. The undifferentiated progenitor cells expressed genes characteristic of both sexual fates, explaining the plasticity of the gonadal cells to differentiate as male or female cell types. The establishment of sex-specific fate in both the germ cells and somatic cells involved activation of some genes; but, importantly, we show that an active repression of genes associated with the alternative pathway is also a characteristic of cell fate commitment. Although germ cell progenitors expressed genes associated with both possible fates, genes characteristic of the male fate were over-represented in the progenitors, giving them a male bias. However, in somatic cell progenitors, which control sex determination, genes associated with the female fate were over-represented. These results suggest an explanation for why the female fate is the developmental default for the gonad, and they advance our understanding of how complex transcriptional networks regulate fate determination during organ development.

## Introduction

Little is known about how the transcriptome of an undifferentiated progenitor is poised between two fates and resolves to one of two stable, differentiated outcomes during the process of cell fate determination. The bipotential gonad is a unique system for studying cell fate decisions during mammalian organ development. In mice, the gonads arise around embryonic day 10 (E10.0). For the first 36 hours the gonad primordia harbor the potential to become testes or ovaries irrespective of genetic sex. The process by which the bipotential gonad adopts the ovarian or testicular fate is known as primary sex determination, and involves a binary fate decision within cells of each gonadal lineage.

The early gonad is composed of four lineages: supporting cells, interstitial/stromal cells, germ cells, and endothelial cells. The determinant of gonadal fate in mammals is the Y-chromosome gene *Sry*. In XY individuals, expression of *Sry* in supporting cell progenitors triggers commitment to a testicular (“male”) fate, whereas the absence of *Sry* expression in XX supporting cells results in ovarian (“female”) development [1], [2]. Supporting cells and interstitial/stromal cells arise within the urogenital ridge from a common mesodermal progenitor, while the primordial germ cells and endothelial cells migrate into the developing gonad [3]–[6]. Despite their distinct origins, cells of each lineage in the early gonad are bipotential progenitors capable of adopting either a male or female fate, which they do in a coordinated manner to form a functional testis or ovary [7]–[9]. Temporal examination of the transcriptomes of these diverse progenitors as they make their parallel, binary fate decisions provides an opportunity to understand how cell fate decisions are made in the context of organ development.

Some gonadal lineages have been studied at the transcriptome level in independent experiments [10]–[15], resulting in the identification of genes that are up-regulated in a sex- or (in some cases) lineage-specific manner. However, the molecular relationship between the somatic lineages (i.e., supporting cells versus interstitial/stromal cells) has never been examined, as these lineages were not separated in previous studies. Previous studies did not fully characterize the undifferentiated progenitors or the temporal sequence for the divergence of the multiple progenitors to their sexual fates. Furthermore, other potentially important transcriptional patterns associated with differentiation and fate commitment, such as the specific transcript depletion previously noted in the *Arabidopsis* root and in early primordial germ cells [16], [17], have not been characterized.

As part of the GenitoUrinary Molecular Anatomy Project (GUDMAP, http://www.gudmap.org/), we undertook a comprehensive transcriptome analysis of the four principle gonadal lineages in XX and XY gonads at three time points, spanning the period from the undifferentiated bipotential stage until the cells adopt sex-specific fates. While this type of comprehensive transcriptome analysis has been performed in other developing systems [16], [18], the relative simplicity of the gonad and the theoretical framework for sex determination allowed us to extend our analysis to test distinct models for the process of cell fate determination, and to evaluate the fit of these models to the theories of sex determination that have been proposed in the past 50 years.

To explore how the cells of the gonad adopt their sex-specific fates, we considered the various theories that have been proposed for gonadal sex determination. It has been suggested that the female fate is the “default” state because expression of *Sry* is required to “divert” the cells to the male fate [19]–[21]. The concept of a female default state originated in the secondary sex determination literature [19], but that language crept into the field of primary sex determination and became a way to conceptualize female gonad development [20], [21]. Others proposed that a female-promoting “Z” gene normally blocks an underlying male developmental program and that Z is itself blocked by *Sry* [22]. Still others have proposed that both the female and male programs require an active switch to initiate differentiation from their initially “bipotential” state (i.e., that there is an “ovary-determining gene” as well as a “testis-determining gene”) [23]. More recently, it has been suggested that the gonad is balanced between the male and female fates by antagonistic signaling pathways [24]–[27].

We wanted to determine whether one of these models could describe the differentiation of the gonadal cells at the level of the transcriptome. Therefore, we re-framed and logically extended these models so that we could test them against our transcriptional data, and we used the concept of lineage priming to do so. Studies suggest that multipotent cells are not a “blank slate”, but rather are “lineage primed” by expressing markers of all potential fates they can adopt [28]–[35]. During their differentiation, multipotent cells repress markers of specific fates that were not adopted while maintaining gene expression associated with the fate that was adopted [28]–[35]. A similar phenomenon has also been observed in the early embryo, where individual blastomeres express transcripts that later become restricted to the specific lineages of the blastocyst [36]–[38]. It is possible for progenitor cells to be equally “balanced” between their multiple fates, expressing similar numbers of genes associated with each alternative differentiated fate. However, the progenitors need not have all differentiation programs equally represented [28]. Instead a progenitor may show “biased priming” if markers characteristic of one of its possible fates predominate, indicating the closer relationship of the progenitor to that fate.

We investigated the transcriptional profiles of the gonadal cell lineages as they differentiate. Interestingly, we identified different variations of biased lineage priming: while germ cells showed male-biased priming, supporting cells showed female-biased priming. This study provides a molecular explanation reconciling the female default and balanced models of sex determination and represents a rich resource for the field. In addition, it affords insight into the mechanisms by which different cell types in a single organ adopt their respective fates.

## Results

### Sorted cell microarrays accurately reflect known gene expression patterns

**Figure 1:**
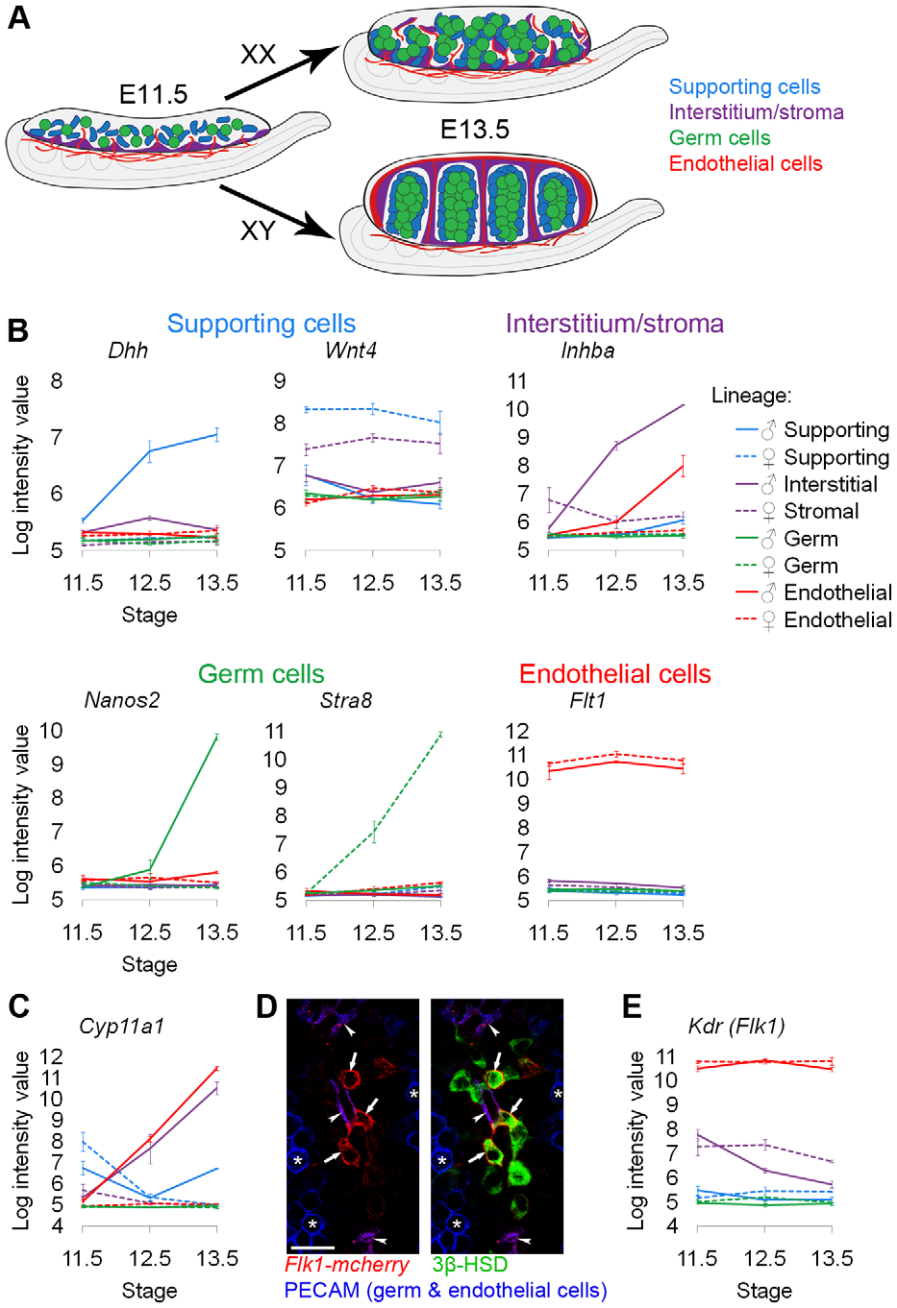
Sorted cell lineages and microarray validation. (A) Illustration of the developing XX and XY gonad with supporting cells (blue), interstitial/stromal cells (purple), germ cells (green), and endothelial cells (red). (B, C, and F) Graphs of the log-transformed, normalized intensity values from the microarrays for control genes known to be specific to each lineage. The color for each lineage is conserved in all figures and matches the illustration (A), with XX (♀) values shown as dashed lines, and XY (♂) values shown as solid lines. The error bars are standard error of the mean (“standard error”) of the log transformed values. The Y-axis scale differs for each graph because each transcript cluster has its own intensity range. (B) The control genes were found in the expected lineage, except for (C) genes characteristic of Leydig cells. Leydig cell genes were highly expressed in both the interstitium (as expected) and the endothelial cell fraction. (D) Immunofluorescence of E13.5 XY gonads with *Flk1-mCherry* (red), PECAM1 (germ and endothelial cells, blue), and 3β-HSD (Leydig cells, green). Arrowheads indicate *Flk1-mCherry* and PECAM1 double positive endothelial cells. Arrows indicate *Flk1-mCherry* positive, PECAM1 negative cells that were positive for 3β-HSD, confirming aberrant reporter expression in some Leydig cells. Asterisks indicate germ cells positive for PECAM1 alone. Scale bar = 25 µm. (E) The XY interstitial cells have very low expression of the endogenous *Flk1* (*Kdr*) transcript at E13.5, supporting our conclusion that the *Flk1-mCherry* transgene is aberrantly expressed in Leydig cells.

We quantified global gene expression in four lineages of the XX and XY developing mouse gonad at E11.5, E12.5, and E13.5. To isolate individual lineages, we utilized mouse lines expressing fluorescent cell-specific markers (Figure 1A, Figure S1). The cells from separately pooled XX and XY gonads were isolated by fluorescence-activated cell sorting (FACS). *Sry-EGFP* [7] and *Sox9-ECFP* [39] were used as markers for supporting cells (see Materials and Methods for a full explanation). XY interstitial cells and XX stromal cells were isolated using *Mafb-EGFP* [40], [41]. The XY interstitial cells are excluded from testis cords and give rise to steroidogenic Leydig cells [41]. The XX stroma is not defined morphologically, but for the purposes of this analysis, is defined as the population labeled with *Mafb-EGFP*. Germ cells were isolated using *Oct4-EGFP* [42], and endothelial cells were isolated using *Flk1-mCherry* [43], [44]. In general, cells were pooled from gonads of multiple embryos on non-inbred genetic backgrounds (see Materials and Methods). RNA purified from each XX and XY cell population was used to measure transcript abundance with Affymetrix Mouse Genechip Gene 1.0 ST Arrays. We produced 3 biological replicates for each population. The data are available in GEO (accession number GSE27715) and at http://www.gudmap.org/. RMA normalized values used in our analysis are provided with the capability to generate an expression graph for any gene, as a user-friendly resource for the community (Dataset S1).

We also isolated cells from the *αSma-EYFP* transgenic mouse [45] with the expectation that this reporter would label a larger population of cells in the interstitium and stroma (Figure S2A). While this population transcriptionally resembled the interstitial/stromal population isolated with the *Mafb-EGFP* line (data not shown), unlike the *Mafb-EGFP* positive cells, the *αSma-EYFP* cells also expressed high levels of *Sry* at E11.5, a gene predicted to be specific to XY supporting cells [46] (Figure S2B). Indeed, E11.5 *αSma-EYFP* cells stained positive for both SRY and SOX9 protein (Figure S2C). Consequently, at least early in development, it appears that *αSma-EYFP* labels a heterogeneous population of cells containing supporting cells as well as interstitial/stromal cells. While this finding may have biological significance, it could also result from leaky expression of the *αSma-EYFP* transgene. Thus, we excluded the *αSma-EYFP* data from this analysis but have made the data available with the rest of the microarrays.

To validate the cell sorting and microarray data, we examined the expression of genes known to be specific to each of the cell populations (Figure 1B). The expression of each control gene was consistent with previous reports: *Dhh* (desert hedgehog) was enriched in XY supporting cells [10], *Wnt4* (wingless-related MMTV integration site 4) was enriched in XX supporting cells [10], *Inhba* (inhibin beta-A) was enriched in the XY interstitium [47], [48], *Nanos2* (nanos homolog 2) was enriched in XY germ cells [49], *Stra8* (stimulated by retinoic acid gene 8) was enriched in XX germ cells [50], and *Flt1* (FMS-like tyrosine kinase 1, VEGFR1) was enriched in endothelial cells [51]. Therefore, the microarrays on the sorted cell populations accurately reflected gene expression patterns that were previously characterized for each of the cell lineages.

An unexpected expression pattern was observed in XY *Flk1-mCherry*-positive cells (Figure 1C). We found that genes expressed in Leydig cells, such as *Cyp11a1* (*Scc*) [52], were enriched in both XY interstitial (containing steroidogenic Leydig cells) and *Flk1-mCherry*-positive “endothelial” populations. This was surprising given that steroidogenic enzymes have not previously been reported in endothelial cells [41]. When we immunostained the *Flk1-mCherry*-positive cells purified by FACS for a Leydig cell marker, we found that Leydig cells were present in this sorted population (data not shown). To investigate the basis for this finding, we stained E13.5 *Flk1-mCherry* gonads with the vascular marker PECAM1 and an antibody against the Leydig cell marker 3β-HSD. We found *Flk1-mCherry*-positive cells that were negative for the vascular marker, but positive for the Leydig marker (Figure 1D). This suggests that the *Flk1-mCherry* transgene was ectopically expressed in some Leydig cells. Because the corresponding *Flk1* transcript (*Kdr*) was expressed at low levels in the XY interstitial cells at E13.5 (Figure 1E), we do not believe that the *Flk1-mCherry*-positive, 3β-HSD-positive cells express endogenous *Kdr/Flk1*. We concluded that the source of the Leydig cell contamination was leaky expression of the *Flk1-mCherry* transgene in Leydig cells. While this result complicated our analysis of the endothelial population, it provided strong evidence that the array data accurately reflected gene expression in the sorted populations.

### Lineage, sex, and stage all influence gene expression

**Figure 2:**
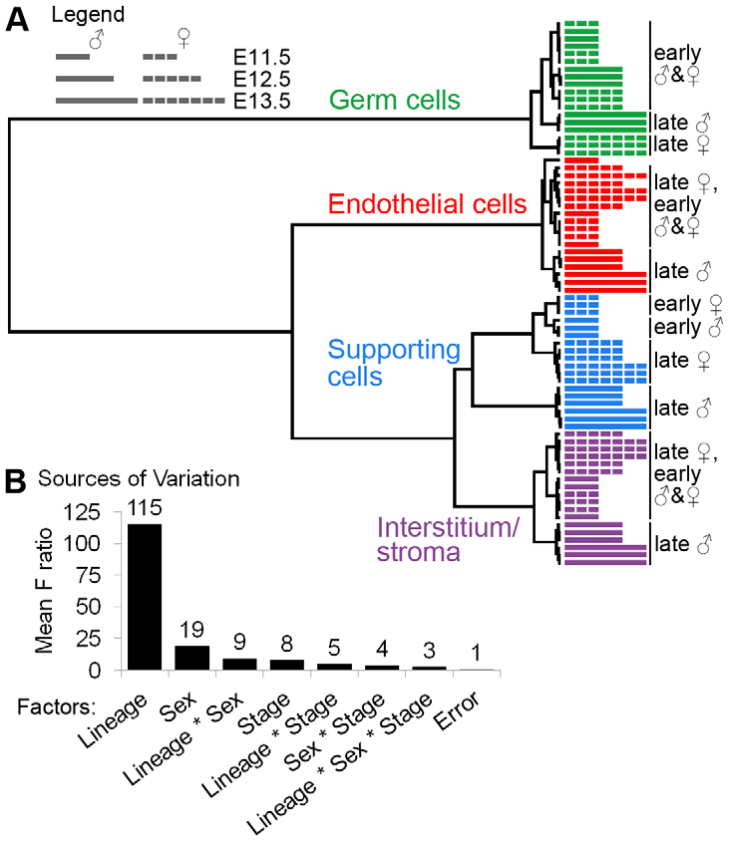
Gene expression was affected by lineage, sex, and stage. (A) Clustering dendrogram of individual microarray samples. The E11.5, E12.5, and E13.5 samples are represented by short, intermediate, and long bars, respectively. The dashed bars indicate XX samples, and the solid bars indicate XY samples. Ward's method with squared Euclidean distance as the distance metric was used. The arrays cluster primarily by lineage, and secondarily by sex and stage. (B) Analysis of the sources of variation confirmed that the primary source of variation is lineage, and secondarily sex and stage.

To investigate the effects of cell lineage, sex, and stage on global gene expression and to validate the consistency of expression measurements in biological replicates, we clustered all 72 individual microarrays based on the expression of transcript clusters that met our inclusion criteria outlined in the Materials and Methods (Figure 2A). With the exception of one E11.5 XY endothelial array (see Materials and Methods), biological replicates, and even samples of different sexes (early XX and XY germ cells) or stages (late XX supporting cells) that were expected to be similar, showed consistent expression patterns as indicated by the tight clustering of those samples (Figure 2A). This again validates the quality of the microarray data.

The dendrogram indicates that cell lineage is the most significant factor affecting gene expression. The same general patterns were observed using other clustering methods (Figure S3A, S3B). This result was confirmed by an analysis of the sources of variation (ANOVA), in which lineage was identified as the most significant factor influencing gene expression variation (Figure 2B, Figure S3C). Interstitial/stromal cells were distinct from supporting cells at all stages, indicating that, despite their shared origin [3], these are separate lineages by E11.5.

Sex and stage were also sources of expression variation in the gonadal cell populations, albeit to a lesser extent than lineage (Figure 2A, 2B). XY and XX supporting cells clustered in distinct groups at E11.5, confirming that these cells embark on their sex-specific differentiation by E11.5 [10], [11]. There was no distinction between the sexes in the other cell types until E12.5 or E13.5. While the late stage XY endothelial cells clustered away from the early XX and XY endothelial cells, this could be due to the sex-specific contamination by Leydig cells. In summary, this analysis confirms the high quality of the data and shows that each lineage is distinct from E11.5 onwards.

### Each lineage has uniquely expressed enriched and depleted genes that provide insight into the biology of the cells

**Figure 3:**
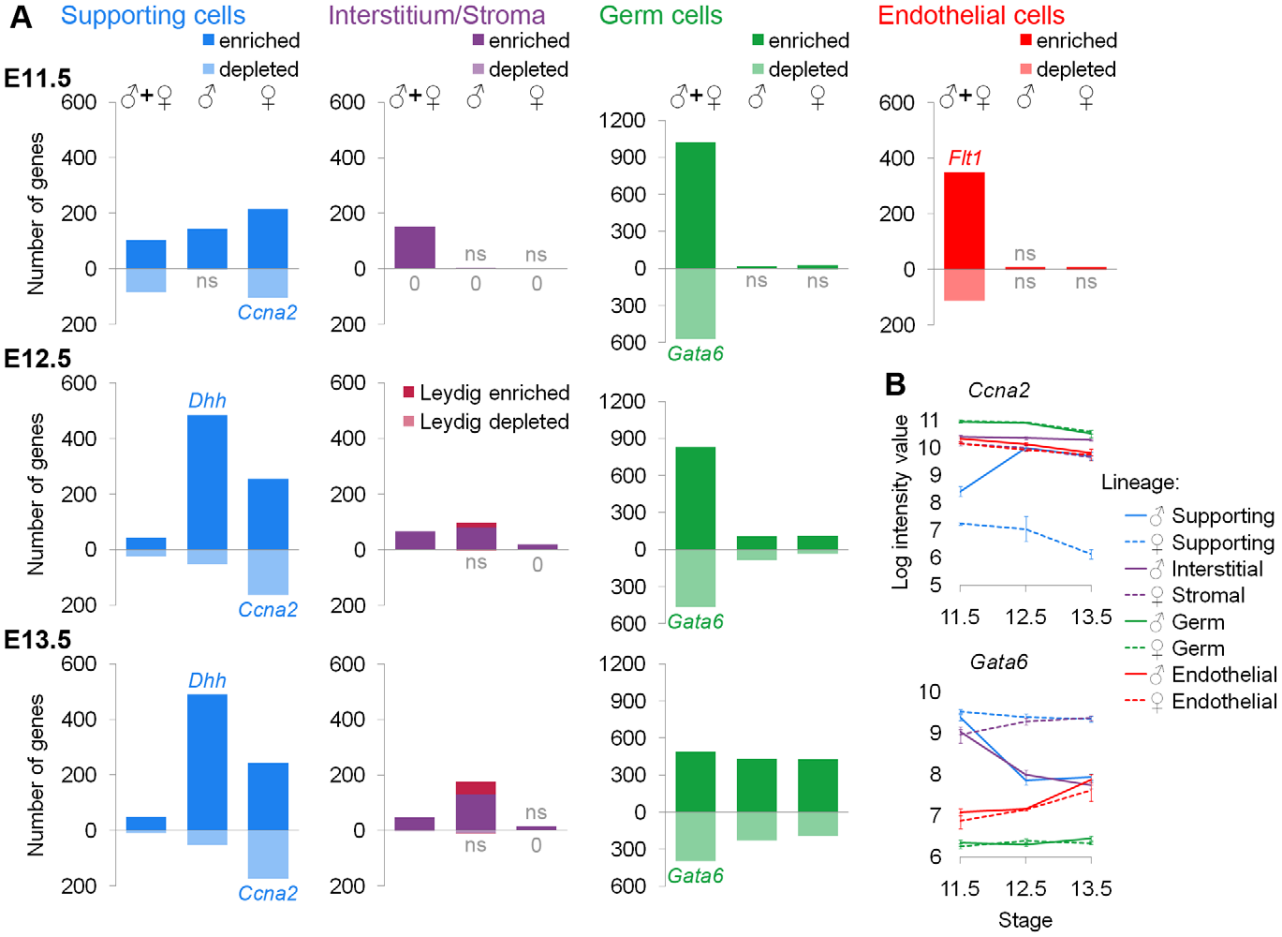
Lineage-specific enriched and depleted genes revealed distinct differentiation programs. (A) Graphs of the number of genes specific to each lineage. The gene lists and permutation tests are provided in Dataset S2. The “♂” and “♀” symbols indicate lineage-specific and sex-specific genes, while the “♂+♀” symbol indicates genes that are lineage-specific and sex-independent. Pale bars below the axis indicate genes that are depleted relative to other lineages. The E11.5 graphs are on the top row, the E12.5 graphs are in the middle, and the E13.5 graphs are on the bottom row. The germ cell Y-axis is scaled to accommodate the larger number of genes specific to this lineage. Leydig cell genes (burgundy) were separately identified by cross-referencing the endothelial and interstitial data and added to the bars for the XY interstitium. Lists with >20% false positives are indicated by “ns”. Lists with no genes are marked with “0”. Some bars also have a colored gene name exemplifying the pattern within that category (the graphs for *Dhh* and *Flt1* appear in Figure 1B). (B) Graphs of the log-transformed, normalized intensity values for genes that are sex-specifically (*Ccna2*) and sex-independently (*Gata6*) depleted. The error bars are standard error. Three lineages showed specific gene depletion in addition to enrichment. Each lineage had transcriptionally distinct progenitors as indicated by “♂+♀” genes at E11.5. Supporting cells were already in the midst of their sex-specific differentiation by E11.5 as indicated by genes in the “♂” or “♀” columns at E11.5, but the other cell types were sexually undifferentiated at E11.5.

To explore the differences between the cell types apparent in the dendrogram, we identified “lineage specific” genes that were specifically enriched and depleted in each lineage relative to the other lineages at each stage. We then determined whether these genes were expressed in a “sex-specific” (expression was different between XY and XX samples) or a “sex-independent” (expression was similar in XY and XX samples) manner. We identified these genes by performing multiple pairwise comparisons on the normalized array values (similar to previously described methods [53]) with a p-value cutoff of 0.05 and a fold change cutoff of 1.5. “Enriched” genes were more highly expressed than in other lineages, and “depleted” genes were less highly expressed than in other lineages. Examples of genes showing these different patterns include the sex-specific enrichment of *Dhh* in XY supporting cells (Figure 1B, Figure 3A), the sex-independent enrichment of *Flt1* in XX and XY endothelial cells (Figure 1B, Figure 3A), the sex-specific depletion of *Ccna2* (cyclin A2) in XX supporting cells (Figure 3A, 3B), and the sex-independent depletion of *Gata6* (GATA binding protein 6) in germ cells (Figure 3A, 3B). A full description of our statistical methods is provided in the Materials and Methods and a list of all genes identified appears in Dataset S2A.

Due to the sex-specific Leydig cell contamination of the endothelial population after E11.5 (Figure 1C–1E), we present the gene lists (Dataset S2A) but not the graphs (Figure 3A) because we could not determine whether sex-specific gene expression was due to an artifact of the contamination or genuine differences in endothelial cells. We deduced a list of Leydig cell genes (Figure 3A, Dataset S2A) by identifying genes specifically over-expressed or under-expressed in both XY endothelial cells and interstitial cells at E12.5 and E13.5 compared to other lineages. These genes were removed from XY endothelial gene lists at E12.5 and E13.5 (Dataset S2A).

To evaluate these lists, we first performed permutation testing (Dataset S2B), and considered those with a false positive rate <20% as acceptable (lists that did not pass this test are marked “ns”) (Figure 3A). Second, we determined whether positive control genes with known expression patterns, such as the expression of *Sox9* in XY supporting cells, were found in the expected lists (Dataset S2A). Third, we interrogated all significant lists in Figure 3A (not “ns” or “0”) for enrichment of BioCarta and KEGG pathways (Dataset S2C) as well as GO terms (Dataset S2D). As evidence of the high quality of the sorted cell microarray data, functional annotation of the enriched/depleted lists identified expected terms for each cell type: germ cell development in the germ cell lists, steroid production in interstitial and Leydig lists, vascular development in the endothelial list, and sex determination in supporting cell lists (Dataset S2C, S2D). All enriched terms for significant lists are provided to facilitate the discovery of new pathways involved in the functions of these cells (Dataset S2C, S2D).

This analysis identified several novel expression features of these cells. For example, all of the lineages had sex-specific and sex-independent cohorts of depleted genes, with the exception of interstitial/stromal cells (Figure 3A). The genes identified as depleted appear biologically relevant based on individual genes and enriched categories of genes. Both XY and XX germ cells repressed *Gata6* (Figure 3B, Dataset S2A), which can drive embryonic stem cells to adopt the extraembryonic endoderm fate [54]. Thus, the repression of *Gata6* may be important for maintaining a totipotent transcriptional state in germ cells. Similarly, *Lef1* became sex-specifically depleted in XY supporting cells (Dataset S2A). *Lef1* can interact with β-catenin in a transcriptional complex downstream of Wnt signaling [55], and this pathway antagonizes aspects of testis development [24], [56]. Thus, the depletion of *Lef1* may be important for maintaining the male supporting cell fate. The sex-specifically depleted genes in XX supporting cells were enriched for cell cycle-related pathways and GO terms (Dataset S2C, S2D). Interestingly, both XX and XY supporting cells are arrested at E11.5 and only XY supporting cells re-enter the cell cycle; XX supporting cells remain non-proliferative [57], [58]. It was previously shown that XX cells express higher levels of cell cycle inhibitors [11] and that cell cycle genes are over-represented in XY supporting cells [15]. However, our data suggest a mechanism of cell cycle arrest involving the active repression of multiple genes important for cell cycle progression in XX supporting cells.

Additionally, we identified transcripts associated with a sexually undifferentiated progenitor cell for each lineage. All cell types had a large number of genes that were lineage-specific and sex-independent at E11.5 (Figure 3A, “♂+♀” category). The identification of shared expression in XX and XY cells demonstrates that there is a sexually undifferentiated progenitor for each lineage with a distinct transcriptional state. This was consistent with previous data suggesting that XX and XY supporting cells have a common origin [7] as well as the clustering results showing that supporting cells and interstitial/stromal cells are distinct lineages by E11.5 (Figure 2A).

Supporting cells exhibited the largest number of sexually dimorphic genes at E11.5 (Figure 3A, “♂” and “♀” categories), in accord with previous evidence that the supporting cell lineage adopts a sex-specific fate early in gonad development and instructs the other lineages as to which fate they should adopt [8], . Although it was clear that supporting cells began sex-specific differentiation by E11.5, XX and XY supporting cells still expressed sex-independent genes. Since the supporting cells are in the midst of their sex-specific differentiation at E11.5, the sex-independent genes likely represent remnants of the sexually-undifferentiated progenitor state. The XX and XY supporting cells adopt their distinct sex-specific states by E12.5.

The other three cell types exhibited few sex-specific genes at E11.5 (Figure 3A, categories “♂” and “♀”), showing that the differentiation of these lineages is delayed relative to that of the supporting cells. Germ cells and interstitial/stromal cells began to adopt lineage-specific, sex-specific transcriptional states by E12.5, a process that was further advanced by E13.5. This was again consistent with the dendrogram (Figure 2A). We identified few lineage-specific genes in the XX stroma at these stages. While some genes were over-expressed in XX stromal cells when compared only to XY interstitial cells, most were also dimorphic in another lineage (Figure S4). This may indicate that the XX stromal cells are delayed in their differentiation and/or closely related to the supporting cell lineage based on shared expression of female-associated genes (like *Irx3* and *Wnt4*; Figure S4).

### Predictions of the gonad differentiation models

These analyses defined the transcriptome shared between XX and XY progenitors for each lineage, and traced the timing of differentiation and acquisition of sex specific fate for each cell type. Informed by this analysis, we investigated whether the transcriptome shared by XX and XY progenitors in the germ cell and supporting cell lineages showed evidence of lineage priming toward the female or male fate. Progenitors that show lineage priming express markers of the various fates into which they can differentiate and subsequently silence genes associated with the fate not adopted as they differentiate [28]–[35].

**Figure 4:**
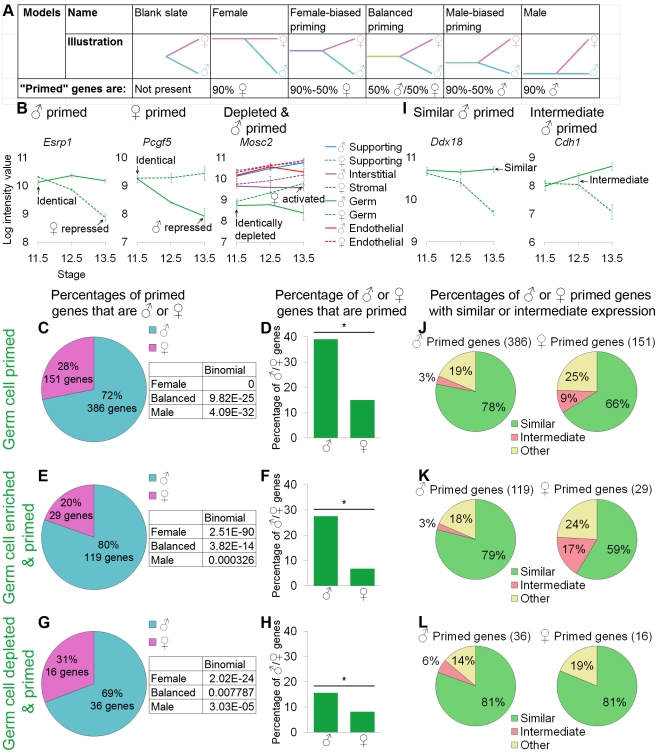
Germ cells showed lineage priming with a male bias. (A) Models tested and their predictions for the relationship between the differentiated cells and their undifferentiated progenitors. (B, I) Graphs of the log-transformed, normalized intensity values. The error bars are standard error. Only the values for germ cells are shown, except in the depleted and primed example where all lineages are shown for comparison (B). (B) *Esrp1* and *Pcgf5* are examples of male- and female-primed genes, and *Mosc2* is an example of a male-primed depleted gene. We used three different methods to identify primed genes: (C, D, and J) all primed genes were considered, (E, F, and K) only primed and lineage-specifically enriched genes were considered, and (G, H, and L) lineage-specifically depleted primed genes were analyzed. (C, E, and G) The percentages of primed genes that were male-primed and female-primed: all methods showed male-biased priming. The boxes contain the p-values from the binomial test with the expected percentages of the extreme models: 90% male genes (“Male”), 50% male and female genes (“Balanced”), and 90% female genes (“Female”) (see A). All of the extreme models were excluded because p-values were <0.05. (D, F, and H) The percentages of male or female genes that were primed. Significance (*) was determined with the hypergeometric test (p-value<0.05). (I) Graphs illustrating two primed genes whose expression in progenitors is “similar” to the differentiated cell in one sex or “intermediate” between the two sexes. (J–L) In all cases, for both sexes, the majority of primed genes were similarly expressed in germ cell progenitors and differentiated cells of one sex. Gene lists and permutation tests are provided in Dataset S4.

Four predominant models have been proposed to account for gonad differentiation that have important historical antecedents in the sex determination literature, and make different predictions as to the “ground state” of gonadal progenitors (Figure 4A). It has been proposed that (1) the female state is a default pathway [19]–[21], (2) a female “Z” gene actively represses the male program [22], (3) both the female and male programs are actively initialized [23], and (4) the gonad is balanced between the male and female fates [24]–[27]. We logically extended and reframed these models in the context of lineage priming so that we could test their applicability to the differentiation of the individual cell types in the gonad at the level of the whole transcriptome.

The “female” model predicts that the transcriptome shared by XY and XX progenitors should be predominately associated with the differentiated female fate (i.e., “female primed”). Conversely, the “male” model predicts that the transcriptome shared by XY and XX progenitors should be predominately associated with the differentiated male fate (i.e., “male primed”). If both the male and female programs are activated *de novo* as progenitors differentiate, those progenitors should be a “blank slate” in that they would not express transcripts associated with either the differentiated male or female cells. Alternatively, the progenitors could fit the “balanced priming” model and express a similar number of both male- and female-associated transcripts at the time when they are poised to adopt either fate. Finally, the progenitors could be primed to adopt either fate, but there could be more genes associated with the female (“female-biased priming”) or the male (“male-biased priming”) fate (Figure 4A).

### Germ cell progenitors are primed with a bias toward the male fate

We analyzed the relationship between XX and XY germ cell progenitors and their final sexually differentiated fates in multiple ways to ensure that our methods did not skew the results. We defined a gene as “primed” if it showed identical levels of expression in XY and XX germ cells at E11.5 that were retained or elevated in one sex and not in the other at E13.5 (Figure 4B, *Esrp1* and *Pcgf5*). This was an unbiased method of identifying genes characteristic of the female and male fates [60]. Similarly, we determined “expression” in the E11.5 sexually undifferentiated progenitors in an unbiased way. We inferred “expression” from the fact that these genes showed down-regulation in one of the sexes. In this approach, all primed genes were analyzed, regardless of whether they were specific to germ cells (Figure 4C, 4D).

In a second approach, we adopted more stringent, but still unbiased, definitions for the set of genes analyzed (Figure 4E, 4F). In this approach, we restricted our analysis to primed genes that were lineage-specifically enriched in E11.5 germ cells and in differentiated E13.5 germ cells of one sex (Figure 3A). Using an analogous method, we also explored depleted and primed genes that were specifically depleted in XY and XX E11.5 progenitors, were activated by one sex, and remained sex-specifically and lineage-specifically depleted in the other (Figure 4B, *Mosc2*, Figure 4G–4H). Gene lists and associated permutation tests are provided (Dataset S4A, S4C).

With all methods tested, germ cells showed a similar pattern indicating male-biased priming. When comparing the percentage of primed genes that were male or female primed, we observed a clear bias toward male genes (Figure 4C, 4E, 4G). We performed a binomial test using the expected values provided with the models (Figure 4A) to determine if we could exclude the extreme models. In all cases, the pattern of genes observed was significantly different from the extreme female and male models as well as the completely balanced priming model (Figure 4C, 4E, 4G). Thus, a male-biased priming model best described the transcriptome in undifferentiated XX and XY germ cell progenitors. This finding was consistent with the clustering dendrogram showing a closer relationship of the undifferentiated early germ cells to the late XY germ cells than to the late XX germ cells (Figure 2A).

We also wanted to ensure that this result was not a statistical artifact of the size of the underlying lists of male and female markers. For example, if the male program contained a larger number of genes than the female program, seeing a male bias in the number of primed genes could reflect the higher relative percentage of male pathway genes, rather than a real priming bias in the progenitor. Thus, we examined the percentage of male and female genes that showed priming (Fig 4D, 4F, and 4H). Again, in all cases, we saw that the same male bias was preserved. Given the large number and percentage (nearly 40% of male germ cell markers) of genes that showed priming (Figure 4C, 4D), the blank slate model could be discarded. Thus, germ cell progenitors showed male-biased priming, including the priming of depleted genes.

Lastly, we were interested in determining whether genes that showed priming were expressed at high or low levels. The expression levels of differentiation markers in the progenitor cells are low in the hematopoietic system [29], but high levels of expression of differentiation markers were observed in progenitors in the early embryo [36]. For our analysis, expression level was defined relative to the differentiated cell. A gene with “similar” expression was expressed in progenitors at a level similar to the level in sexually differentiated cells maintaining expression (Figure 4I, *Ddx18*), analogous to the high expression observed in the early embryo [36]. A gene with “intermediate” expression was expressed in progenitors at a level between the levels observed in the two sexes (Figure 4I, *Cdh1*), analogous to the low expression observed in hematopoietic cells [29]. We analyzed the expression level for genes identified as primed by each method (Figure 4J–4L).

A majority of both male and female primed genes were similarly expressed in the undifferentiated germ cell progenitors and the sexually differentiated cells, regardless of how the set of primed genes was defined or whether enriched or depleted genes were considered (Figure 4J–4L). Thus, not only did germ cells show male-biased priming, but the progenitors frequently expressed these primed genes at the same level as the sexually differentiated cells.

We analyzed the lists of genes that exhibited a primed expression pattern (including all primed genes), and were regulated in the same way (similar expression in the progenitor and sexually differentiated cells, Figure 4J) for enrichment of GO terms (Dataset S4D). The genes primed toward the male germ cell fate showed a strong enrichment for categories related to RNA biology, such as RNA binding (Dataset S4D). This is consistent with the previously reported importance of gene control at the RNA level during germ cell development, especially in the male [49], [61], [62].

All the data indicate that the germ cells are primed with a male-bias, and that these primed genes are generally expressed at a similar level in the progenitor and differentiated cell.

### Supporting cell progenitors are primed with a bias toward the female fate

**Figure 5:**
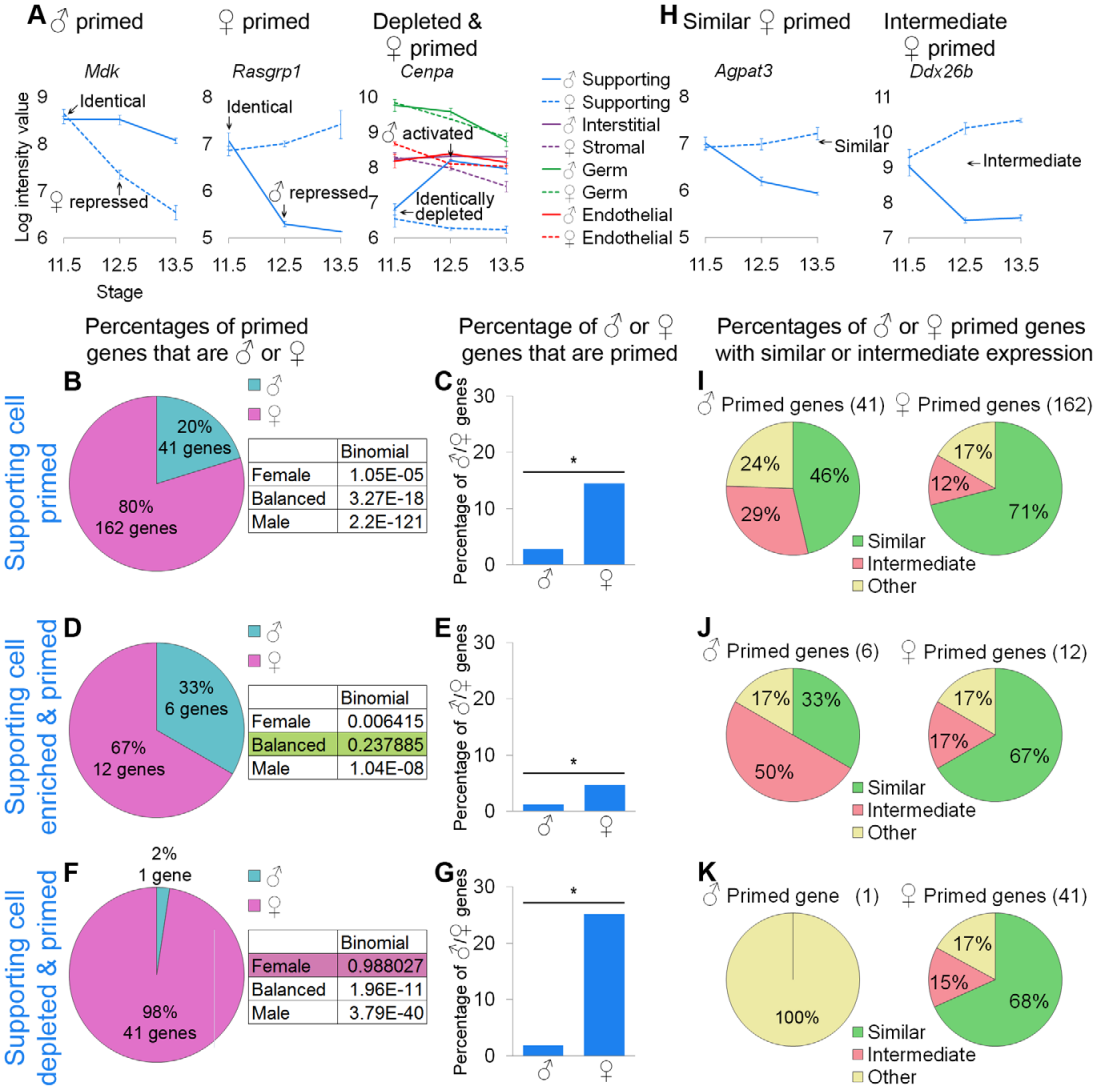
Supporting cells showed lineage priming with a female bias. (A and H) Graphs of the log-transformed, normalized intensity values of genes. The error bars are standard error. Only the values for supporting cells are shown, except in the depleted and primed example where all cell types are shown. (A) *Mdk* and *Rasgrp1* are examples of male- and female-primed genes and *Cenpa* is an example of a female-primed depleted gene. As in the germ cell analysis, we examined all primed genes (B, C, and I), primed and lineage-specifically enriched genes (D, E, and J), and primed and lineage-specifically depleted genes (F, G, and K). (B, D, and F) The percentages of primed genes that were male-primed and female-primed. The boxes contain the p-values from the binomial test with the expected percentages of the extreme models. (B) Using the first method, all of the extreme models could be excluded because they had a p-value<0.05. (D and F) However, using the second and third methods, the balanced and female models could not be excluded, respectively. (C, E, and G) Nevertheless, examining the percentage of male or female genes that were primed, all methods showed a significant (*) bias toward the female pathway, as determined by the hypergeometric test (p-value<0.05). Taken together, the data supported female-biased priming. (H) Graphs illustrating two primed genes, whose expression in the progenitor is “similar” to the differentiated cell of one sex, or “intermediate” between the two sexes. (I–K) The female-primed genes were predominantly similarly expressed, but the male-primed genes showed more variability. Gene lists and permutation tests are provided in Dataset S4.

We also examined the relationship between supporting cell progenitors and their sexually differentiated states. We used the same method and tested the same models as with germ cells, but the end point of the analysis for supporting cells was E12.5 because their transcriptome changed little between E12.5 and E13.5 (Figure 3A, Figure S4, Dataset S2A, Dataset S3A). As in the germ cell analysis, we defined a gene as “primed” if it showed identical levels of expression in XY and XX supporting cells at E11.5, which were retained or elevated in one sex and not in the other by E12.5 (Figure 5A). The same variations of the analytical methods used for germ cells were also used to identify supporting cell-specifically enriched and depleted primed genes (Figure 5A). The gene lists, permutation test results, and GO terms are provided (Dataset S4B-S4D).

The pattern in supporting cells was less consistent than the pattern observed for germ cells, but was indicative of female-biased priming. Examining all of the primed genes, there was a clear female bias both for the primed genes as a percentage of the priming program (Figure 5B) and the primed genes as a percentage of the XY and XX sexually dimorphic genes (Figure 5C). When restricting our analysis to the genes specifically enriched in supporting cells (Figure 5D, 5E), we observed a similar female bias in the percentage of XX and XY genes that were primed (Figure 5E), but the bias in the percentage of primed genes associated with the male and female pathways was not sufficient to exclude the balanced priming model (Figure 5D). This is likely a statistical artifact due to the small number of primed genes and the difference in size between the E12.5 XX and XY enriched supporting cell gene lists (Figure 3A). The genes specifically depleted in supporting cells showed a strong female bias (Figure 5F, 5G), such that the female model could not be excluded (Figure 5F). All of these analyses suggested female-biased priming, consistent with the clustering showing the closer relationship between the less differentiated early supporting cells and the late XX supporting cells compared to the late XY supporting cells (Figure 2A).

We also determined if the primed genes showed similar or intermediate expression in undifferentiated supporting cell progenitors (Figure 5H). For the female-primed genes, we consistently observed that most genes were similarly expressed (Figure 5I–5K). Expression levels of male-primed genes were more variable (Figure 5I–5K), which again could be due to the small number of genes analyzed. Nevertheless, supporting cell progenitors expressed at least some of the primed genes at levels similar to the sexually differentiated supporting cells.

**Figure 6:**
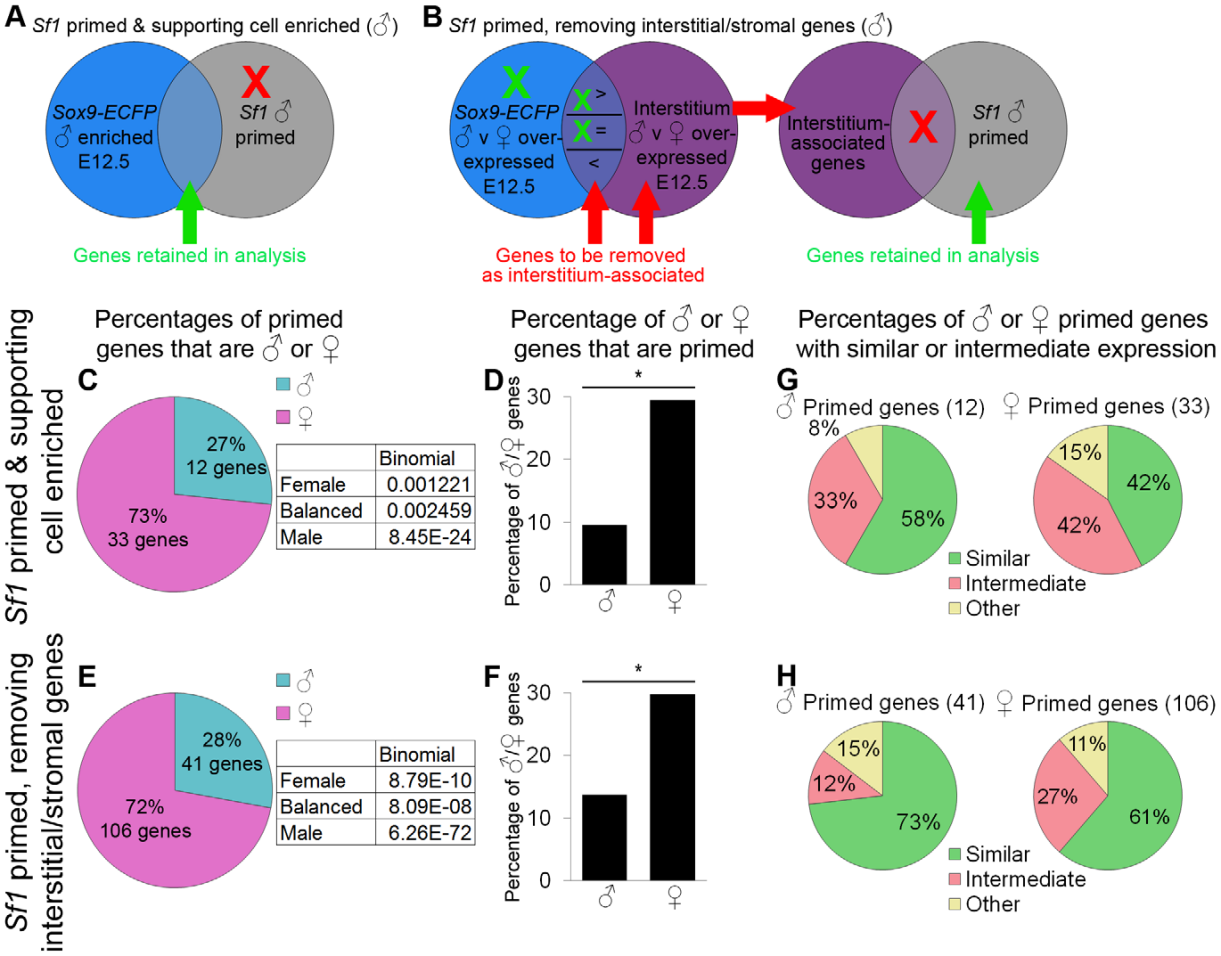
Data from sorted *Sf1-EGFP* cells also supported female-biased priming for supporting cells. (A–B) Graphical illustrations of the genes included in our analysis of priming in the *Sf1-EGFP* data. Because the *Sf1*-positive population is a mixture of lineages, we used two methods to identify the primed genes associated with supporting cells. XY cells are illustrated in this example, but the same operations were also performed for XX cells. (A) “*Sf1* primed and supporting cell enriched” genes were both male-primed in the *Sf1-EGFP* data (comparing E11.0 and E12.5) and lineage-specifically enriched in our XY *Sry-EGFP/Sox9-ECFP* purified supporting cells at E12.5. Red indicates genes being removed from the analysis, and green indicates genes being retained. (B) For the “*Sf1* primed, removing interstitial/stromal genes”, we removed genes associated with the interstitial/stromal cells at E12.5 (i.e., sexually dimorphic in the interstitium/stroma) from the *Sf1-EGFP* primed genes. Genes that were expressed sexually dimorphically in both the interstitial/stromal cells and the supporting cells were removed only if expression was higher in the interstitial/stromal cells than in the *Sry-EGFP/Sox9-ECFP* supporting cells. The *Sf1-EGFP* primed genes that were enriched in the *Sry-EGFP/Sox9-ECFP* supporting cells (C, D, and G) and those that were identified by removing interstitial/stromal genes (E, F, and H) were analyzed separately. (C and E) The percentages of primed genes that were male-primed and female-primed. Both methods showed a female bias. The boxes contain the p-values from the binomial test with the expected percentages of the extreme models, and all extreme models could be rejected as having a p-value<0.05. (D and F) The percentage of male or female genes that were primed showed a significant (*) bias toward the female pathway, as determined by the hypergeometric test (p-value<0.05). (G and H) However, primed genes in both sexes were predominantly expressed at similar levels in progenitors and E12.5 supporting cells of one sex. While supporting cell progenitors have a female bias, they also express some markers of the male pathway at levels similar to male supporting cells at E12.5. Gene lists and permutation tests are provided in Dataset S5.

However, the XX and XY supporting cells were already expressing sexually dimorphic genes by E11.5 (Figure 3A) and therefore were not fully undifferentiated at the start of our analysis. With the reporters available to us, collection of a pure population of progenitors from an earlier stage was not feasible. To determine whether our results were affected by the differentiation process already in progress, we reanalyzed a publically available microarray time course on sorted *Sf1-EGFP* cells from the urogenital ridge that included earlier time points than were collected here [11] (Figure 6, Figure S5, Dataset S5). While the *Sf1-EGFP* reporter allows for the collection of earlier time points, it also labels a mixed population that includes at least supporting cells and interstitial/stromal cells [11], [52]. This is concerning because different cell types can have different priming patterns (Figure 4, Figure 5). Therefore, we identified genes from the *Sf1-EGFP* data set that showed priming, and then restricted our analysis to only those genes whose expression patterns were associated with the supporting cell lineage as defined by our data set.

Using the same approach as in the analysis of the *Sry-EGFP/Sox9-ECFP* sorted supporting cell progenitors, we identified primed genes within the *Sf1-EGFP* undifferentiated progenitor population. To limit the contribution of genes from interstitial/stromal cells, we set the end point of the analysis at E12.5. E11.0 *Sf1-EGFP* cells were selected as the starting point for the analysis presented in Figure 6 because it was closest to the sexual divergence point of the primed genes, although the analysis from E10.5 is also provided (Figure S5, Dataset S5B).

While lists of all primed genes identified in the *Sf1-EGFP* cells are provided (Dataset S5A), we limited our analysis to only those genes associated with supporting cells. To define this gene set, we used two different methods utilizing our purified supporting cell (*Sry-EGFP/Sox9-ECFP*) and interstitial/stromal cell data as a reference. First, we used a rigorous threshold for inclusion and retained only those genes that were also found to be lineage-specifically and sex-specifically enriched in our E12.5 XX or XY *Sry-EGFP/Sox9-ECFP* supporting cells (Dataset S2A; Figure 6A, 6C, 6D, 6G). In a second approach, we used less rigorous criteria and removed genes sexually dimorphic in the interstitial/stromal cells (Figure 6B, 6E, 6F, 6H; Dataset S5A, S5C). Genes that were sexually dimorphic in both the *Sry-EGFP/Sox9-ECFP* supporting cells and the interstitial/stromal population were only removed if they were expressed at higher levels in interstitial/stromal cells than in supporting cells.

Regardless of the method used, these data also supported female-biased priming of the supporting cells. Most of the primed genes were female, although the progenitors expressed some male genes as well (Figure 6C–6F, Dataset S5A). Many of these genes were also similarly expressed (Figure 6G, 6H; Dataset S5A). This analysis is therefore consistent with the findings from the *Sry-EGFP/Sox9-ECFP* data. To investigate the overlap in these data sets, we determined whether similar transcripts were identified as primed in both the *Sf1-EGFP* and *Sry-EGFP/Sox9-ECFP* data sets (Figure S6). While a small percentage of the genes primed in the *Sf1-EGFP* cells were also identified as primed in the *Sry-EGFP/Sox9-ECFP* data, a larger proportion were already sexually dimorphic by E11.5, although indications of priming could be observed in some of the expression patterns (Figure S6). This analysis was also consistent with previous findings showing that individual genes we identified as primed were expressed in the supporting cell progenitors and then became sex-specific (*Dax1/Nr0b1*, *Wnt4*, *Sox9*, and *Cbln4* [26], [63]–[66], Dataset S4B, Dataset S5A). Thus, the analysis of these two independent data sets produced consistent results for individual genes and reached the same overall conclusion that the supporting cells are primed with a female-bias.

Because nearly 30% of female genes showed priming in the *Sf1-EGFP* data (Figure 6D, 6F), the blank slate model can be rejected. The E11.5 *Sry-EGFP/Sox9-ECFP* progenitors showed a lower percentage of primed transcripts (Figure 5C, 5E) than the E11.0 *Sf1-EGFP* progenitors (Figure 6D, 6F), which may be explained by the fact that the *Sry-EGFP/Sox9-ECFP* supporting cell progenitors were already partially sexually differentiated by E11.5 (Figure 3A).

## Discussion

A comprehensive understanding of organogenesis requires systems-level knowledge of transcriptional network dynamics underlying cell differentiation. By performing a microarray analysis on sorted cell populations in the fetal mouse gonad over the course of sex-specific differentiation, we quantified the transcription dynamics of diverse cell types as they build one of two different organs from similar pools of progenitors. This study provided an expression resource for the field of gonad development, but more importantly, it characterized the features of the biological system that could only be appreciated at the whole transcriptome level. By examining the system as a whole, we obtained new insights into patterns of cell fate determination and lineage commitment.

### Insights from whole transcriptome characterization of multiple gonadal lineages

**Figure 7:**
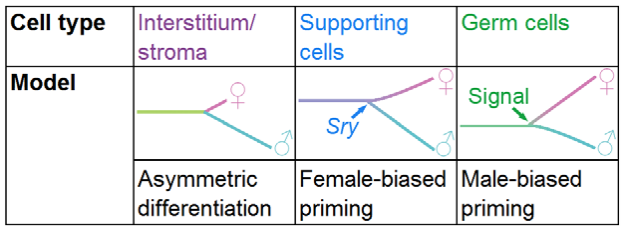
Models of differentiation for the different gonadal lineages. The interstitial/stromal cells differentiate asymmetrically over the time period examined, as we detected few genes specific to the XX stroma by E13.5, whereas, the XY interstitial population acquired a larger set of lineage-specific genes. Supporting cells are primed with a female bias. The natural progression of the primed state may be to adopt the female differentiated state, but in the presence of *Sry* the cells repress the female program and adopt the male fate. Conversely, germ cells are primed with a male bias. An extrinsic signal may be required from the mesonephros to induce the adoption of the female fate; otherwise, germ cells adopt the male fate.

We characterized the transcriptomes of undifferentiated progenitors and analyzed their transition to sexually differentiated cells. All four lineages analyzed (including interstitial/stromal cells) have a sexually undifferentiated progenitor cell with a distinct transcriptome. Although we detected some overlapping sexually dimorphic expression patterns that may have biological significance between the supporting cells and interstitial/stromal cells (Figure S4), these lineages have transcriptionally distinct progenitors at E11.5 (Figure 3A). However, the differentiation of the interstitial and stromal cells is sexually asymmetric over this time period (Figure 7). Whereas the XY interstitium expressed lineage-specific transcripts, there were few lineage-specific transcripts in XX stromal cells even at E13.5 (Figure 3A). Thus, the XX stroma may not fully differentiate until after E13.5. The XX stroma also showed overlapping sexually dimorphic expression with XX supporting cells, in part due to pathways downstream of widespread Wnt signaling in the ovary (Figure S4).

We also provided global transcriptional evidence that the supporting cells are the first cell type in the gonad to adopt a sex-specific fate (Figure 3A), as predicted by previous experiments [10], [11], [46], [59]. While there are some gene expression differences between E12.5 and E13.5, the supporting cells appear to have adopted their sex-specific fates by E12.5 (Figure 3A, Figure S4, Dataset S2, Dataset S3). The sex-specific differentiation of interstitial/stromal cells and germ cells began at E12.5, when the supporting cells had essentially completed their differentiation process (Figure 3A).

### Lineage- and sex-specific transcriptional depletion in the differentiating gonad

For supporting cells, germ cells, and endothelial cells, our methods were sensitive enough to detect lineage-specific transcript depletion that could be sex-specific or sex-independent (Figure 3A). Given that other studies have also reported specific gene depletion [16], [17], this likely represents a common regulatory logic in the transcription network of differentiating cells. For example, unique cell fate specification in the sea urchin involves the repression of widely-expressed genes to “lock-down” the selected fate [67]. We detected the lineage-specific repression of transcription factors likely involved in specifying alternative fates in both germ and supporting cells (i.e., *Gata6* and *Lef1*, Figure 3, Dataset S2A). We also found evidence for lineage-specific repression of genes that regulate cell behavior. The transcriptome of XX supporting cells is characterized by the sex- and lineage-specific repression of cell cycle genes (Figure 3, Dataset S2), which is correlated with the failure of XX supporting cells to reenter the cell cycle [57], [58]. A similar phenomenon was reported in senescence and DNA damage arrest [68], [69], indicating this may be a widely-used mechanism of cell cycle arrest.

Depleted genes also showed evidence of lineage priming in progenitors with similar patterns as genes expressed and primed (Figure 4G, 4H; Figure 5F, 5G), particularly in germ cells. The association between components of the network that are expressed and repressed suggests that both are important for specifying cell fate and may be co-regulated as parts of the same transcriptional program [67].

Contrary to our findings in the other lineages, we did not detect lineage-specifically depleted genes in the XY interstitial or XX stromal cells. However, there may be heterogeneity within this population that masks repression characteristic of any one subfraction. Since the purity of the germ and supporting cell populations were likely important for detecting depletion, the ability to isolate distinct populations within the interstitium/stroma may be necessary to do the same for this population.

### A role for priming in the bipotential supporting cell lineage

We determined that the supporting cell progenitors are primed with a female bias, indicating that both male and female genes are expressed in the progenitor, but that the female program is over-represented (Figure 7). This female-biased priming model bridges the more recent evidence for a balance in the gonad between the male and female fates [24]–[27] and the classic theory of the female “default” state [19]–[21]. Although we found genes characteristic of both the male and female programs in supporting cell progenitors, the over-representation of the female program in progenitors explains why the female fate is the “default” state in the absence of *Sry*.

The female fate may be the “default” state because the over-represented female program of supporting cell progenitors is self-sustaining without additional inputs and leads to silencing of the alternative testis pathway. The high level expression of primed genes may make the primed state in the progenitors unstable. Whereas, low-level expression of fate determinates has been associated with a stable primed state, the expression of determinates at high levels has been associated with instability of the primed state [70]. As priming with high level expression has also been noted in the early embryo [36], this may be a common developmental mechanism to ensure that development progresses and does not become stalled. Thus, this unstable primed state would naturally lead to female differentiation in the absence of an intervention from *Sry*. Under these circumstances, an ovary-determining gene, as proposed by Eicher and Washburn [23], would not be necessary.

Differences in the strength of the female bias may explain differences between humans and mice with respect to the effects of mutations in individual genes in the female pathway. For example, mutation of *RSPO1* in humans resulted in female-to-male sex reversal [71]. However, deletion of *Wnt4*, *Rspo1*, or β-catenin in the mouse did not result in full female-to-male sex reversal [25], [66], [72], [73]. The female bias in mice may be sufficiently robust to rebound from the loss of any one of these genes. Conversely, a lower level of female-biased priming in humans could allow deletion of just one key factor in the female program to disrupt female development.

Bipotential supporting cells show priming toward the male and female fates (Figure 5, Figure 6). A previous study identified a female subnetwork in E11.5 XY gonads [27]. We identified many of these genes as female-primed (*Wnt4*, *Fst*, *Rspo1*, *Dapk1*, *Pld1*, *Actr6*, and *Dock4*, Dataset S5A). The expression of female genes in XX and XY supporting cell progenitors could be consistent with a female transcriptional state in the progenitors that is repressed by the activation of *Sry* in XY cells rather than by the concept of male and female priming.

However, the coexpression of male genes in both XX and XY supporting cell progenitors strongly supports the idea that these cells are primed to adopt either of their potential fates. These genes likely represent a male subnetwork operating in the early XX and XY cells independent of *Sry*, as *Sry* is not present in the XX cells. This work provides a molecular explanation for the concept of bipotentiality characterized by the coexpression of both male and female transcripts in the XX and XY supporting cell progenitors.

The mechanism of priming remains to be determined. It has been speculated that priming is a byproduct of open chromatin [29], [33], [70]. Bivalent chromatin has been reported in embryonic stem cells at loci expressed at low levels [74]. However, this is inconsistent with our findings in the gonad where primed genes in bipotential supporting cell precursors tended to be expressed at high levels. Other studies in the laboratory are aimed at investigating the state of chromatin at primed loci.

While priming may be important to establish a bipotential state, mounting evidence suggests that repression of the primed genes associated with the alternative sex is important for supporting or maintaining cell fate commitment. Genes important for testis (*Sox9* and *Dmrt1*) [75]–[77] and ovary (*Wnt4*, *Fst*, and *Rspo1*) [25], [66], [78] development were identified as expressed in XX and XY supporting cell precursors and repressed in the opposite sex (Dataset S4B, Dataset S5A). Over-expression of *Sox9* is known to result in female-to-male sex reversal [79]. Ectopic activation of the downstream target of WNT4, β-catenin, can reverse differentiation of XY supporting cells and trigger their differentiation as female cells [56]. Thus, the repression of genes associated with the opposite sex (which we identified in these experiments) may be as essential to the cell fate decision as the genes that are expressed.

### Priming during germ cell development

The testicular-biased primed state of germ cell progenitors was surprising because germ cell fate is determined by the somatic environment [8]. Germ cells that enter an ovarian environment initiate meiosis (the female fate), while germ cells that enter a testis environment undergo mitotic arrest (the male fate) [80]. Historically, entry into meiosis was thought to be the default state for germ cell differentiation [8], [81]–[84]. However, the weight of current evidence indicates that meiosis (the female fate) is the result of an external inducing signal produced in the mesonephros and specifically degraded in the testis by *Cyp26b1* [85]–[89]. While there is some evidence for a signal promoting the male fate [90]–[94], this signal may act by antagonizing the female-promoting signal [85], [91] and/or providing a permissive environment for male germ cell development [90].

The male-biased transcriptome of germ cell progenitors is consistent with a male developmental “default” state in the absence of the female-promoting signal (Figure 7). Interestingly, in both supporting and germ cells, the dominant fate-determining signal is associated with the fate under-represented in the progenitor's transcriptome (Figure 7). *Sry* expression in XY supporting cells is required to stabilize the male program and repress the female program. Similarly, the external female signal initiates the meiotic program in germ cells and represses the alternative male program. XY germ cells adopt the female fate if *Cyp26b1* activity is eliminated (resulting in the presence of the meiosis-inducing signal), even in an otherwise male environment [85], [86]. Our priming model suggests that the over-represented male program requires only subtle reinforcement from the somatic environment. On the other hand, the under-represented female program cannot be stabilized without its instructive cue, but once that input is received, it is able to suppress the male program.

One reason why E13.5 XY germ cells share more transcriptional features with the progenitor than XX germ cells may be due to their maintenance in a more stem cell-like state (Dataset S4A) [95]. However, XY germ cells at E13.5 are not identical to the sexually undifferentiated germ cell progenitors at E11.5. Specifically, by E13.5, XY germ cells have repressed genes associated with the female germ cell program (Figure 4), which may explain why, even when put into a female environment after E11.5, XY germ cells can no longer adopt the female fate [8], [81].

### Priming during differentiation

This study revealed previously unknown systems-level aspects of the differentiation of two critical cell types during gonad development, with implications for other developing cells. Supporting and germ cells arise from different embryonic origins and respond to different cues during their terminal differentiation, and yet both show priming. Priming may be a common feature of differentiation from multipotent progenitors at all levels, as it has now been identified in the early embryo [36], multipotent hematopoietic cells [29],bone marrow mesenchymal stem cells [28], germ cells, and somatic gonadal cells.

However, each priming program appears surprisingly lineage specific. Even the cells within the gonad do not share a common bias in their priming programs (Figure 7). Priming may limit the developmental potential of cells by preparing them to respond in a unique manner to the same signals used throughout development. Only certain avenues of differentiation are available while others are closed [28], [30], [60]. For example, male supporting cells and male germ cells exposed to similar *Fgf9* signals adopt different fates [24], [91], [92], [96] because they have different underlying transcriptional networks that prepare the cell to respond differently. *In vivo*, a supporting cell progenitor cannot become a germ cell because the required transcriptional avenues are not available. The ability to induce pluripotent cells from differentiated cells *in vitro* may be related to the ability to return the cell to a primitive primed state, where many avenues of differentiation are open.

We identified priming patterns using simple, yet flexible, statistical methods that can be applied to any microarray time course on a single purified cell population isolated immediately preceding and following differentiation. While we were able to analyze the priming of lineage-specific enriched and depleted transcripts to validate our results (Figure 4, Figure 5), the results were similar regardless of the method used. Because having other cell types for comparison is not required, this method can be broadly applied to other systems exploring differentiation.

Systems biology entails the use of both whole genome analysis and molecular genetic approaches to inform each other [97]. While studies disrupting the function of individual genes have clearly identified critical components of the system, they are unlikely to be sufficient on their own to fully elucidate the combinatorial interactions within the complex transcriptional network governing organ development. Recent studies show that developmental transcriptional networks are highly buffered and contain redundant factors, suggesting that many important network players may not have a developmental phenotype when disrupted [27], [98]. While some transcriptional profiles uncovered in this study may have no functional relevance, others likely contribute to robustness of the system and allow it to rebound from perturbation.

In conjunction with the traditional functional studies examining individual genes, our understanding of gonad organogenesis (and development in general) is facilitated by a whole system view of the process as this approach reveals novel phenomena that cannot be identified by studying single genes. This analysis leads to many new and exciting hypotheses related to the role of priming in the differentiation of gonadal cells and provides new insight into the processes of cellular differentiation and lineage commitment.

## Materials and Methods

### Mice

All animals were maintained and experiments were conducted according to DUMC-IACUC and NIH guidelines, based on existing protocols. We used six different transgenic mouse lines with fluorescent reporters: *Sry-EGFP* [Tg(Sry-EGFP)92Ei] (sex determining region of Chr Y) [7], *Sox9-ECFP* (SRY-box containing gene 9) [39], *Mafb-EGFP* [Mafb^tm1Jeng^] (a gift from S. Takahashi; v-maf musculoaponeurotic fibrosarcoma oncogene family, protein B) [40], *αSma-EYFP* (a gift from J. Lessard; the official gene name of *αSma* is *Acta2*; actin, alpha 2, smooth muscle) [45], *Flk1-mCherry* [Tg(Kdr-mCherry)1Medi] (a gift from M. Dickinson; the official gene name of *Flk1* is *Kdr*; kinase insert domain protein receptor) [43], [44], and *Oct4-EGFP* [Tg(Pou5f1-EGFP)2Mnn] (the official gene name of *Oct4* is *Pou5f1*; POU domain, class 5, transcription factor 1) [42]. In most cases, males from these lines were crossed to CD-1 females (an outbreed line from Charles River), and gonads from multiple embryos were generally pooled to reduce the impact of strain variation. All of the males were homozygous for the marker, with the exception of *Mafb-EGFP* males. This line is a targeted insertion of GFP into the *Mafb* locus, which results in a *Mafb* mutant when homozygous. *Mafb-EGFP* embryos collected in this study were therefore heterozygous for *Mafb*; however, we know of no defects in gonad development in *Mafb* heterozygotes (data not shown). To increase the fluorescence intensity for both the *αSma-EYFP* (maintained on an FVB/CD-1 mixed line) and *Flk1-mCherry* (maintained on the outbreed CD-1 line) reporters, homozygous males were crossed to homozygous females.

*Sry-EGFP* and *Sox9-ECFP* reporters were used to collect supporting cells. In the *Sry-EGFP* line, the *Sry* promoter drives expression of GFP in cells competent to activate the *Sry* promoter in both XX and XY gonads. This labels the supporting cell lineage in both sexes, but because the transgene lacks the SRY open reading frame, transgenic XX gonads express no SRY protein and develop as normal ovaries [7]. While *Sry-EGFP* expression persists in XX supporting cell precursors through E13.5, expression of the transgene is reduced in XY supporting cell precursors after E11.5 in our hands (data not shown), similar to endogenous *Sry* expression [99]. Therefore, *Sry-EGFP* was used to isolate supporting cells from XX and XY samples at E11.5, and XX E12.5 and E13.5 samples. *Sox9-ECFP* was used to sort XY supporting cells at E12.5 and E13.5. All cells that later express *Sox9* can be lineage traced from *Sry*-positive cells [46]. *Sox9* is also the direct downstream target of SRY [100]. This *Sox9-ECFP* reporter specifically labels XY supporting cells (Figure S1). Thus, we did not expect that the use of *Sox9-ECFP* would affect our analysis of the supporting cells. We validated that this was the case by ensuring the genes we identified in the *Sox9-ECFP* population substantially overlapped those identified in a previous study using the *Sry-EGFP* reporter [10], and that some genes continued to be similarly expressed in the XX *Sry-EGFP* and XY *Sox9-ECFP* supporting cells (data not shown).

### Collection of gonadal lineages

Timed matings were performed, with the day the vaginal plug was detected considered E0.5. Embryos were collected at E11.5, E12.5, and E13.5. For the *Mafb-EGFP* line, only GFP-positive embryos were used for sorting. The sex of the gonad is obvious by eye at E12.5 and E13.5. The E11.5 embryos were genotyped to determine the sex as previously described using primers to detect *Kdm5c* (X chromosome) and *Kdm5d* (Y chromosome) [27], [101].

To collect gonadal cells, the urogenital ridge and dorsal aorta were removed, and the gonad/mesonephric complex was isolated. In most cases, the gonad was separated from the mesonephros. However, for the *Oct4-EGFP* sorts, the gonad was left attached because *Oct4* expression is highly specific to germ cells (data not shown). For the E11.5 *Flk1-mCherry* sorts, only the anterior and posterior portions of the mesonephros were removed by cutting at a 45° angle from the end of the gonad. The gonad vasculature arises from a plexus in the mesonephros [5]; thus, the gonadal and a portion of the mesonephric endothelial cells represent one population. This procedure retained the mesonephric plexus while removing most of the vasculature associated with the mesonephric ducts. At E12.5 and E13.5, the mesonephros was removed completely for the *Flk1-mCherry* sorts.

XY and XX gonads were separately pooled from one or more litters and incubated in 250 µl 0.25% Trypsin EDTA (Gibco #25200) at 37°C for 5–10 minutes. The trypsin was removed and replaced with 400 µl PBS with or without 4 µl RNase-free DNase (Promega #M6101). The tissue was dissociated, and the cells were passed through a strainer (BD Falcon #352235). FACS was performed by the Duke Comprehensive Cancer Center Flow Cytometry Shared Resource. The positive fraction was pelleted, the liquid supernatant was removed, and the cells were immediately frozen at −80°C.

### Preparation of samples and arrays

Generally, cells from multiple embryos were pooled. RNA was extracted from over 100,000 cells to as few as 10,000 cells using the RNeasy Micro kit (Qiagen #74004) following the manufacturer's instructions for “Cells.” However, the protocol was started at step 2 (disruption with RLT), and β-ME was not added. The cells from multiple sorts were pooled during disruption with RLT (if necessary), step 3 (homogenization) was skipped, and in step 10 three RPE washes were performed.

Samples were prepared for the Affymetrix Genechip Mouse Gene 1.0 ST Arrays (#901168) using the Nugen WT-Ovation Pico RNA Amplification System (#3300), WT-Ovation Exon Module (#2000), and the Encore Biotin Module (#4200), following the manufacturer's instructions. For purification following the Pico and Exon kits, the Qiagen QIAquick PCR Purification Kit (#28104) was used following the instructions provided by Nugen. Fragmented and labeled product was submitted to the Duke Institute for Genome Sciences and Policy Microarray Facility for hybridization and reading.

We ran a total of 91 arrays. This included the arrays on our five sorted cell types at the three time points with separate XX and XY samples in biological triplicate as well as one array on a whole P1 mouse RNA sample(a gift from S. Potter) for normalization across GUDMAP.

### Immunofluorescence

Samples were fixed, stained, and imaged as whole gonads with the mesonephros attached as previously described [102]. Some samples were first processed through a methanol series and stored at −80°C prior to rehydration and staining [103]. Primary antibodies used were: anti-3β-HSD (Santa Cruz sc-30820, 1∶100 in samples processed through methanol; TransGenic Inc. KO607, 1∶500; K. Morohashi generously provided a non-commercial antibody before the TransGenic Inc. antibody was available), anti-PECAM1 (BD Pharmingen 553370, 1∶250), anti-SRY (a gift from P. Koopman and D. Wilhelm), and anti-SOX9 (a gift from P. Koopman and D. Wilhelm). Secondary antibodies used included Alexa 647- and 488- conjugated secondary antibodies (Molecular Probes, 1∶500) and Cy3- and Cy5-conjugated secondary antibodies (Jackson ImmunoResearch, 1∶500). DAPI (Sigma-Aldrich) was used to label nuclei.

### Initial array processing and analysis

The .cel files were processed with Partek Genomics Suite version 6.5 (6.11.0207) by RMA with quantile normalization and median polish summarization at the transcript cluster (gene) level. Probes were adjusted for GC content and probe sequence. The data were transformed into log base 2. We removed all transcript clusters that did not have a cross hybridization category of 1 (perfect match) in the Affymetrix annotation, that did not have a gene symbol, or that did not have a log base 2 normalized expression value >6 in at least two out of three replicates of at least one sample. The genes that passed this filtering step, and only these genes, were used in subsequent analyses.

In the case of the analysis of the *αSma-EYFP* cells (Figure S2), this initial processing of the arrays included all 91. However, for all other analyses, we used data generated by processing only 72 of our arrays because the *αSma-EYFP* and P1 whole mouse data were not included. These processed data on the 72 arrays were used in all portions of the analysis and are provided as a resource for the community (Dataset S1).

Partek Genomics Suite was used to generate the hierarchical clustering dendrograms and perform the ANOVA sources of variation analysis (Figure 2, Figure S3). The clustering methods used are described in the figure legends. One of the E11.5 XY endothelial samples was somewhat of an outlier in the clustering. This may be due in part to its processing, which resulted in unusually low (but still adequate) yield after the amplification with the Pico kit. However, we do not believe this compromised the sample as it still clustered with endothelial cells.

### Pairwise comparisons used to identify genes of interest

This analysis was performed at the level of the transcript cluster (gene), but some genes have multiple transcript clusters. Thus, the lists of transcript clusters may include multiple entries for the same gene. For graphical display of the numbers of genes identified (Figure 3, Figure 4, Figure 5, Figure 6, Figure S4, Figure S5, Figure S6), each gene was counted only once (i.e., duplicates were removed). However, in tables, all transcript clusters are shown (i.e., duplicates are not removed) (Dataset S2, Dataset S3, Dataset S4, Dataset S5).

To identify genes of interest, we adopted a simple and flexible method using multiple or single pairwise comparisons between samples (analogous to methods used previously [53]). The same p-value and fold change cutoffs were used throughout. In all cases where we identified a difference between samples, we used a p-value cutoff of 0.05, and a fold change cutoff of 1.5 for each comparison. A gene was deemed to be identically expressed in two samples if the p-value was>0.05 and the fold change was between −1.5 and 1.5. When multiple pairwise comparisons were done, the intersection of the multiple lists generated was taken as the genes of interest.

To identify sex-specifically and lineage-specifically enriched genes (using E12.5 XY supporting cells as an example), we used the following pairwise comparisons with the above cutoffs:

1. The gene was more highly expressed in XY supporting cells than XX supporting cells at E12.5 (i.e., sex-specific expression).
2. The gene was more highly expressed in XY supporting cells than the XY interstitium, germ cells, and endothelial cells at E12.5 (XY supporting cells versus XY interstitium, XY supporting cells versus XY germ cells, etc.) (i.e., lineage-specific expression).

The intersection of these multiple lists were the genes considered enriched in XY supporting cells at E12.5 (Figure 3A). Because the XY supporting cells were not compared to other XX lineages (e.g., XX germ cells), genes could be identified as enriched in both the XY supporting cells and a different XX lineage. Similar comparisons were also used to identify sex- and lineage-specifically depleted genes, but a gene was deemed “depleted” when its expression was higher in all other cell lineages than the E12.5 XY supporting cells in the example.

To identify sex-independent and lineage-specifically enriched genes indicative of sexually undifferentiated progenitor expression (Figure 3A), we used the following pairwise comparisons (using the E11.5 supporting cells as an example):

1. The gene was more highly expressed in XY supporting cells than the XY interstitium, germ cells, and endothelial cells at E11.5 (i.e., lineage-specific expression among XY cells).
2. The gene was more highly expressed in XX supporting cells than the XX stroma, germ cells, and endothelial cells at E11.5 (i.e., lineage-specific expression among XX cells).
3. The gene was identically expressed in XX and XY supporting cells at E11.5 (i.e., identically expressed in progenitors).

Again, similar comparisons were used to identify the lineage-specifically depleted genes, but a gene was deemed “depleted” when its expression was higher in all other cell lineages than the E11.5 XY and XX supporting cells in the example. Occasionally, multiple transcript clusters for a gene may behave differently. In this case that gene may be identified in multiple lists. For example, different *Myo9a* transcript clusters were identified as sex-specifically enriched in E11.5 XX supporting cells and sex-independently enriched in E11.5 XX and XY supporting cells (Dataset S2A). The different behavior of the different transcript clusters in the gene could be caused by off-target probe binding or alterative splicing.

Leydig cell genes were a special case because both our sorted interstitial and “endothelial” cells contained Leydig cells (Figure 1C–1E). To identify Leydig cell genes (Figure 3A), using E13.5 as an example, we used the following pairwise comparisons:

1. The gene was more highly expressed in the XY interstitium than XY supporting cells and germ cells at E13.5 (i.e., lineage-specific expression).
2. The gene was more highly expressed in XY endothelial cells than XY supporting cells and germ cells at E13.5.
3. The gene was more highly expressed in the XY interstitium than the XX stroma at E13.5 (i.e., sex-specific expression).
4. The gene was more highly expressed in XY endothelial cells than XX endothelial cells at E13.5.

Depleted genes were identified similarly, but a gene was deemed “depleted” when its expression was higher in all other cell lineages than the E13.5 XY interstitium and endothelial cells in the example. The genes identified as E13.5 Leydig cell genes were removed from the E12.5 and E13.5 XY endothelial cell gene lists (Dataset S2A). In all cases, we used the E13.5 Leydig cell lists to remove the maximum number of genes associated with Leydig cells. Some of these genes were also identified in the XY interstitial lists, and they appear in both lists of identified genes (Dataset S2A), but the overlapping genes were removed from the Leydig cell bar for the graphical depiction (Figure 3A).

Permutation testing was done to estimate the false discovery rate in the gene lists. The array data from the samples being used in the generation of a gene list (in the first example above: XY supporting cells, XX supporting cells, XY interstitium, XY germ cells and XY endothelial cells; all at E12.5) were permuted. The series of operations was run on the permuted columns, and the number of genes generated from each permutation was stored. The permutations were performed 200 times, and the mean number of genes was used to compute the false discovery rate. In all cases, we considered a false discovery rate of 20% or less as acceptable (Dataset S2B). Most lists actually had a much lower false discovery rate. In any case where some of the genes were removed from the list, such as the removal of Leydig genes from endothelial cell lists, this operation was ignored in the permutation tests.

The lists of transcript clusters for the lists considered significant were inputted into DAVID (http://david.abcc.ncifcrf.gov/) [104], [105] to identify pathway and GO term enrichment. The full list of transcript clusters used in the analysis (Dataset S1A) was inputted as the background. We included all KEGG and BioCarta pathways as well as GO_FAT molecular function (MF) and Biological Process (BP) terms with a p-value>0.05 (Dataset S2C, S2D).

Primed genes were identified by multiple methods (Figure 4, Figure 5). In general, we defined a gene as primed when it was expressed in the progenitor, then repressed by one sex and maintained or activated by the other sex. To identify all male-primed genes, using germ cells as an example, we used the following pairwise comparisons:

1. The gene was identically expressed in XX and XY germ cells at E11.5 (i.e., identically expressed in progenitors).
2. The gene was more highly expressed in XY than XX germ cells at E13.5 (i.e., specific to XY cells).
3. The gene was more highly expressed in XX germ cells at E11.5 than at E13.5 (i.e., this gene is repressed in XX cells).
4. If the gene was also more highly expressed in XY germ cells at E11.5 than at E13.5, it was removed (i.e., genes showing differential repression were eliminated).

In the second analysis, we used more stringent criteria to define genes characteristic of the progenitor cells at E11.5 and differentiated cells at E13.5 by incorporating information on lineage-specific expression. To identify the enriched and primed genes, we used the above comparisons in addition to requiring that:

1. The gene was more highly expressed in XY germ cells at E11.5 than XY supporting cells, interstitium, and endothelial cells at E11.5.
2. The gene was more highly expressed in XX germ cells at E11.5 than XX supporting cells, stroma, and endothelial cells at E11.5.
3. The gene was more highly expressed in XY germ cells at E13.5 than XY supporting cells, interstitium, and endothelial cells at E13.5.

Finally, we also wanted to explore the possibility of depleted gene priming. Continuing with the example of XY germ cells, we used the following pairwise comparisons to identify depleted and primed genes:

1. The gene was identically expressed in XX and XY germ cells at E11.5 (i.e., identically expressed in progenitors).
2. The gene was more highly expressed in XY supporting cells, interstitium, and endothelial cells at E11.5 than XY germ cells at E11.5 (i.e., lineage specific repression).
3. The gene was more highly expressed in XX supporting cells, stroma, and endothelial cells at E11.5 than XX germ cells at E11.5.
4. The gene was more highly expressed in XX than XY germ cells at E13.5 (i.e., remains repressed in XY cells).
5. The gene was more highly expressed in XY supporting cells, interstitium, and endothelial cells at E13.5 than XY germ cells at E13.5 (i.e., lineage-specific repression).
6. The gene was more highly expressed in XX germ cells at E13.5 than at E11.5 (i.e., this gene is activated in XX cells).
7. If the gene was also more highly expressed in XY germ cells at E13.5 than at E11.5, it was removed (i.e., genes showing differential activation were eliminated).

The same methods were used to analyze priming in the supporting cells, but E12.5 was used as the end point of the analysis. Permutation tests were run on all of these lists of primed genes.

We then examined these primed genes in two ways. First, we compiled all genes identified as male or female primed and determined the percentage associated with each sex (Figure 4C, 4E, 4G; Figure 5B, 5D, and 5F). We used a binomial test for the different extreme models to determine whether priming showed a sex-specific bias. A one-tailed test was used for the “female” and “male” models, which were defined as predicting that 90% of the genes were female-primed or male-primed, respectively. A two-tailed binomial test was used for the balanced model, which predicts 50% male and 50% female genes. Any model resulting in a p-value<0.05 was excluded. If all models were excluded, an intermediate model was selected.

To ensure that this result was not a statistical artifact of the size of the underlying lists of male and female markers, we also displayed primed genes as a percentage of the total “male” or “female” genes (Figure 4D, 4F, 4H; Figure 5C, 5E, 5G). These male and female genes were determined in different ways to account for differences in the method of defining primed genes. When identifying all primed genes, the list of all testis genes included everything identified in step 2 alone of the process for generating the primed genes. For the enriched and primed genes, testis genes were required to meet the requirements of step 2 and 7. For the depleted and primed genes, testis genes were required to meet steps 4 and 5. A 2×2 contingency table (with the actual numbers of genes, not percentages) and a two-tailed hypergeometric test were used to determine if there were a significant difference in the percentage of primed genes in the male and female programs. A p-value<0.05 was considered significant. A significant p-value meant that sex and priming were not independent variables and that there was a bias in the representation of the two programs in the progenitor cells.

We further characterized the primed genes identified by each method based on their expression level. We divided these into two categories: similar and intermediate expression relative to the expression in the differentiated cells (Figures 4J–4L, Figure 5I–5K). For similarly expressed genes, using XY germ cells as an example, the gene was required to be primed and identically expressed in XY germ cells at E11.5 and E13.5. For intermediate expression genes, the gene was required to be primed and more highly expressed in XY germ cells at E13.5 than E11.5 (or more highly expressed at E11.5 for depleted and primed genes). Any gene that did not fall into one of these categories, or had transcript clusters that fell into both similar and intermediate categories, was counted as “other.” If a gene had transcript clusters that fell into the defined similar or intermediate categories and others that did not fall into either, the gene was still counted as similar or intermediate. GO term enrichment for all of the primed genes with similar expression for both supporting and germ cells was determined using DAVID as described above.

### *Sf1-EGFP* array analysis

To examine earlier expression in supporting cells, we reanalyzed previously generated microarray data from *Sf1-EGFP* sorted cells [11] (raw data available at http://www.ebi.ac.uk/arrayexpress/browse.html?keywords=Nef&expandefo=on). Because these data used a different array format (Affymetrix Mouse Genome 430 2.0 arrays), they were analyzed separately. The .cel files were processed with Partek Genomics Suite in the same manner as our own, and the same criteria were used to remove probe sets. The only difference was that rather than using the cross-hybridization category, the annotation grade was obtained from NetAffx (http://www.affymetrix.com) [106], and only probes with unambiguous A and B grade annotation were retained. Control probes were also removed.

We used the same method to identify and analyze primed genes as described above. This analysis used the *Sf1-EGFP* data at E11.0 (or E10.5) and E12.5 (Figure 6, Figure S5, Dataset S5). The priming analysis was limited to probes associated with the supporting cells by two methods, although the list of all primed genes is provided (Dataset S5A, S5B). First, we examined only genes that were lineage-specifically enriched in our XX or XY E12.5 *Sry-EGFP/Sox9-ECFP* data (Figure 3A, Dataset S2A) and *Sf1-EGFP* primed (Figure 6A, 6C, 6D, 6G; Figure S5A, S5B, S5E). Because different arrays were used, we compared between the two datasets using the gene symbol. If the gene symbol for an *Sf1-EGFP* primed gene was also found in the lineage-specific *Sry-EGFP/Sox9-ECFP* lists, the gene was retained in the analysis. Second, we removed the genes associated with the interstitial/stromal cells at E12.5 from the *Sf1-EGFP* primed genes (Figure 6B, 6E, 6F, 6H; Figure S5C, S5D, S5F). Using XY cells as an example, we identified the genes to remove using the following pairwise comparisons and removal steps:

1. We identified genes more highly expressed in XY interstitial cells than XY supporting cells at E12.5.
2. We identified genes more highly expressed in XY supporting cells than XX supporting cells and also XY interstitial cells than XX stromal cells, then removed the genes identified in step 1.
3. We identified all genes more highly expressed in XY interstitial cells than the XX stromal cells, and removed all of the genes remaining in step 2. This list contains genes sexually dimorphic in the interstitial cells and not the supporting cells, and genes sexually dimorphic in both the interstitial/stromal cells and the supporting cells if expression was higher in the interstitial/stromal cells.

The lists of the genes identified for removal is provided in Dataset S5C. Again, the comparison between the two data sets was based on the gene symbol: gene symbols found in the interstitial/stromal-associated lists were removed from the *Sf1-EGFP* primed list.

To calculate the percentage of male and female genes showing priming (Figure 6D, 6F; Figure S5B, S5D), we used the same method used in analyzing all primed genes in our own data. However, in addition to being more highly expressed in one sex at E12.5, the gene was also required to pass the appropriate filter used for identifying supporting cell-associated genes.

## Supporting Information

Dataset S1 RMA normalized values and intensity value graphing. We provided an excel file with (A) the log-transformed RMA normalized intensity values used in our analysis and (B) the graphic format used to display gene expression in each cell type over time. The user can copy any row from (A) with values into row 2 of (B) (yellow) to generate the graph for that gene. Only transcript clusters included in the analysis are provided, and the grounds for removing transcript clusters are provided in the Materials and Methods.
(XLSX)

Dataset S2 Lineage-specific gene lists, permutation tests, pathway enrichment, and GO term enrichment. (A) Gene lists generated by multiple pairwise comparisons, graphically illustrated in Figure 3A. The lists are arranged in order of lineage, stage, enrichment or depletion, and sex. Genes are in alphabetical order. (B) Results of permutation tests for all the gene lists displayed in Figure 3A. Separate entries are listed for the median and mean number of transcript clusters and individual genes found in the permutation tests. The number of genes in the actual list and the percentage of false positives based on the mean number of genes are also provided. (C) The lists of KEGG and BioCarta pathways enriched with a p-value<0.05 in each list with a false discovery rate <20%. (D) The list of biological process (“BP”) and molecular function (“MF”) GO terms enriched with a p-value<0.05 in each significant list. In both cases, the lists are arranged in order of lineage, stage, sex, and enrichment or depletion. The terms in the list are ranked from lowest to highest p-value.
(XLSX)

Dataset S3 Lists of sexually dimorphic genes, overlapping expression, and permutation tests. (A) Gene lists generated by a single pairwise comparison between XY and XX cells of a lineage graphically illustrated in Figure S4A. Lists of all sexually dimorphic genes in each lineage at each stage are provided first, followed by lists of genes sexually dimorphic in multiple lineages. Genes are in alphabetical order. The gene lists are arranged by stage, sex, and lineage. When transcript clusters for a single gene behaved differently, in all cases the gene was counted in the highest overlapping category. For example, if a gene had one transcript cluster sexually dimorphic in only one lineage and another that was dimorphic in two lineages, it was counted as being dimorphic in both lineages. “§” next to a gene name indicates the gene was counted as “♀ E13.5 over-expressed in all cell types” (the triple overlap in the Venn diagram in Figure S4A) even though it appeared in other categories (*Syngap1* and *Myo9a*). (B) Results of permutation tests for the single pairwise comparisons. (C) Lists of genes that were sexually dimorphic in only two lineages, expressed identically in both lineages, and enriched in these two lineages compared to the remaining two lineages (Figure S4E). Genes are in alphabetical order. The gene lists are arranged by lineage, sex, and stage. The gene considered in the triple overlap (*Syngap1*) is again marked by the § and removed from the analysis.
(XLSX)

Dataset S4 Germ cell and supporting cell primed gene lists, permutation tests, and GO term enrichment. (A and B) Gene lists generated by multiple pairwise comparisons to identify germ cell (A) and supporting cell (B) primed genes, graphically illustrated in Figure 4 and Figure 5. Genes are in alphabetical order. The gene lists are arranged by method of identifying primed genes, and sex. The genes with similar and intermediate expression are provided after each list of primed genes. “§” next to a gene name indicates its transcript clusters appeared in both the similar and intermediate expression lists, and so was counted in Figure 4 and Figure 5 as “Other”. (C) Results of permutation tests for the lists of primed genes. (D) The lists of biological process (“BP”) and molecular function (“MF”) GO terms enriched with a p-value<0.05 in all of the primed genes with similar expression (Figure 4J, Figure 5I). The lists are arranged in order of lineage and sex. The terms in the lists are ranked from lowest to highest p-value.
(XLSX)

Dataset S5 *Sf1-EGFP* primed gene lists and permutation tests. (A and B) Gene lists generated by multiple pairwise comparisons, graphically illustrated in Figure 6 and Figure S5. Genes are in alphabetical order. The gene lists are arranged by the method of selecting supporting cell primed genes, and sex. The list of all primed genes identified in the *Sf1-EGFP* data without limiting the list to genes associated with supporting cells, “all”, are also provided. Lists of similar and intermediate expression primed genes follow each list of primed genes (except for the first). Lists are provided starting the priming analysis at both E11.0 (A) and E10.5 (B). (C) Gene lists of interstitial/stromal associated genes that were used to remove interstitial/stromal genes from the priming analysis as illustrated in Figure 6B. (D) Lists of genes illustrated in Figure S5G that were primed at E10.5 or E11.0 and became sexually dimorphic 12 hours later. Genes are in alphabetical order. (E) Lists of genes illustrated in Figure S5H. Of the genes that were primed at E10.5 or E11.0 and remained similarly expressed in XX and XY cells 12 hours later, we identified genes that showed a significant and similar change in expression in both XX and XY cells. Genes are in alphabetical order. (F) Lists of genes illustrated in Figure S6A that were primed in the *Sf1-EGFP* and *Sry-EGFP/Sox9-ECFP* data, were primed in the *Sf1-EGFP* data and dimorphic in the *Sry-EGFP/Sox9-ECFP* data, and a subset of these E11.5 dimorphic genes that showed indications of previous priming in the *Sry-EGFP/Sox9-ECFP* cells. Genes are in alphabetical order.
(XLSX)

Figure S1 Lineage-specific fluorescent tags used for FACS. Images of E13.5 XY and XX gonads with DAPI (blue) and each fluorescent marker used: *Sox9-ECFP* and *Sry-EGFP* (cyan) labeling supporting cells, *Mafb-EGFP* (purple) labeling the interstitial/stromal cells, *Oct4-EGFP* (green) labeling germ cells, and *Flk1-mCherry* (red) labeling endothelial cells. Scale bar = 100 µm.
(TIF)

Figure S2 *αSma-EYFP* labeled a heterogeneous population containing supporting cell precursors. (A) Images of E13.5 XY and XX gonads with DAPI (blue) and *αSma-EYFP* (pink) labeling the interstitial/stromal cells. (B) Graphs of the log-transformed, normalized intensity values. The error bars are standard error. The *Sry* transcript is expressed at similarly high levels in both XY supporting cells and *αSma-EYFP* cells at E11.5, and declines rapidly in both cell types. Expression of *Sry* is lower in the *Mafb-EGFP* cells. However, the pattern seen with *Sry* did not hold true for most supporting cell markers: *Sox9* is expressed at a lower level in both *αSma-EYFP* and *Mafb-EGFP* cells than in supporting cells. (C) SRY and SOX9 proteins are also present in *αSma-EYFP* cells. Antibodies against SRY (red) and SOX9 (blue) co-label *αSma-EYFP* (green) cells. Cells with *αSma-EYFP* and SRY alone are indicated with arrows, whereas cells with *αSma-EYFP*, SRY, and SOX9 are indicated with arrowheads. Scale bar = 25 µm. This suggests that *αSma-EYFP* is expressed in a heterogeneous population of early gonadal cells containing supporting cell progenitors.
(TIF)

Figure S3 Alternative methods showed generally similar patterns indicating the importance of lineage, sex, and stage. Clustering dendrograms of the individual arrays generated using (A) Average linkage with Euclidean distance as a distance metric and (B) Complete linkage with Pearson's dissimilarity as a distance metric. Consistent with Figure 2A, the arrays cluster primarily by lineage, and secondarily by sex and stage. The largest differences were in the relationship of the somatic populations to each other, although the same clusters could always be identified. (C) Examining the sources of variation with the median F ratio shows a similar pattern to the mean F ratio (Figure 2B) with the primary source of variation being lineage.
(TIF)

Figure S4 Overlap of genes that are sexually dimorphic in at least one lineage. (A) The number of genes over-expressed (“sexually dimorphic”) in XY or XX cells of each lineage at each stage. Genes were identified by a single pairwise comparison between XX and XY cells for each lineage at each stage (Dataset S3A). Many more genes are identified here than in Figure 3A because additional pairwise comparisons were performed to restrict the analysis to genes that showed lineage-specificity in Figure 3A. The area proportional Venn diagrams were generated using Venn Diagram Plotter v1.4.3740 from PNNL and OMICS.PNL.GOV (http://omics.pnl.gov/software/VennDiagramPlotter.php). The sizing of the Venn diagrams relative to each other is approximate. Endothelial cells were not analyzed. The numbers shown indicate the number of genes exclusively in each portion of the diagram (except for E11.5 for which the total number of genes dimorphic in the supporting cells is shown). Lists marked “ns” had a false positive rate >20% (Dataset S3B). The overlapping areas on the Venn diagrams indicate genes sexually dimorphic in multiple lineages. Most genes dimorphic in the XX stroma were also dimorphic in another lineage. (B–D) Graphs of the log-transformed, normalized intensity values. The error bars are standard error. Endothelial cell values are not shown. (B) Many genes sexually dimorphic in multiple lineages were over-expressed in one of the lineages, as was the case for *Sox9*. However, this could be explained by the low and variable contamination expected after FACS. To address this issue, we used antibody stains of sorted cells to estimate that the XY E13.5 germ cells had <1% contamination with supporting cells, but the XY E13.5 interstitium was more variable and had between 1% and 15% supporting cell contamination (data not shown). Therefore, patterns similar to *Sox9* were not further analyzed. (C–D) However, not all genes sexually dimorphic in multiple lineages had a pattern consistent with low level contamination. (C) *Axin2*, a Wnt/β-catenin transcriptional target gene [107], was highly expressed in the three XX cell types examined, indicative of widespread Wnt signaling in the ovary. (D) Similarly, *Irx3* (Iroquois related homeobox 3), a gene known to be ovary-specific [108], showed convergent expression in the XX stroma and supporting cells. We identified more genes with a pattern similar to *Irx3* as sexually dimorphic in only two lineages, expressed identically in both lineages, and enriched in these two lineages compared to the remaining two (Dataset S3C). Genes sexually dimorphic in all three lineages were not analyzed. There were few genes meeting these criteria in the overlap between the germ cells and other lineages (Dataset S3C), but (E) more genes met these criteria in the overlap between interstitial/stromal cells and supporting cells (Dataset S3C). The XX stroma in particular expressed several transcripts at similar levels to the XX supporting cells, although the populations are not identical as the XX supporting cells have many more lineage-specific transcripts (Figure 3A). Some of these shared transcripts are downstream of *Wnt4* signaling in the ovary (*Calb1*, *Fgfr2*, *Irx3*, *Sema3a*, and *Tkt*) [109], and *Wnt4* itself showed this pattern. Thus, at least some of these similarities may be attributable to widespread Wnt signaling in the ovary, as reported by *Axin2* expression in all 3 lineages (C).
(TIF)

Figure S5 E10.5 *Sf1-EGFP* primed genes generally supported female-biased priming, but the E11.0 analysis was more informative. The analysis of the *Sf1-EGFP* primed genes (beginning the analysis at E10.5 and comparing to *Sf1-EGFP* cells at E12.5) was also limited to genes enriched in the *Sry-EGFP/Sox9-ECFP* supporting cells at E12.5 (A, B, and E) or to those genes identified by removing interstitial/stromal genes (C, D, and F). (A and C) The percentages of primed genes that were male-primed and female-primed. The first, but not the second, method showed a female bias. The boxes contain the p-values from the binomial test with the expected percentages of the extreme models. The balanced model can be rejected with the first (A), but not the second (C), method. (B and D) Examining the percentage of male or female genes that were primed similarly showed a significant (*) bias toward the female pathway, as determined by the hypergeometric test (p-value<0.05), for the first (B), but not the second (D), method (ns). (E and F) The primed genes for both sexes are predominantly similarly expressed in progenitors and E12.5 differentiated cells. However, E10.5 may not be the appropriate starting point for the priming analysis. (G) No primed genes (identified by removing interstitial/stromal genes) became dimorphic between E10.5 and E11.0 (“0”). Thus, starting the analysis at E11.0 does not result in the loss of any information. Starting at E11.0 is also preferable because it is closer to the divergence point. (H) Between E10.5 and E11.0, 98% of the primed genes were identically expressed in XX and XY samples at both E10.5 and E11.0, and 76% of the primed genes were identically expressed in XX and XY samples at both E11.0 and E11.5 (data not shown). Of these genes, 16% were changing expression level in the same way (see I) in both XX and XY cells between E10.5 and E11.0, whereas only 4% fell in this category between E11.0 and E11.5. This difference was significant (*), as determined by the hypergeometric test with a p-value<0.05, and affects how primed genes are called. This problem is illustrated in (I) by the graphs of the log-transformed, normalized intensity values from *Sf1-EGFP* cells (black) of two genes identified as primed at E10.5, but not at E11.0. *Tpx2* showed significant identical changes in expression in XX and XY cells between E10.5 and E11.0. A similar pattern was observed in many other genes that did not reach significance, such as *Ppil5*, and so this pattern may be more pervasive than indicated by the number meeting the significance thresholds shown in H. This type of expression pattern confounds the analysis when we compare expression at E10.5 to E12.5 to identify primed genes. This may be caused by changes in the number of supporting cell progenitors, or alternatively, the continued transcriptional development of the progenitors between E10.5 and E11.0 may establish the priming program immediately preceding sexual divergence. For these reasons, we chose to start the analysis at E11.0. The gene lists for these analyses are provided in Dataset S5.
(TIF)

Figure S6 Overlap of primed genes between the *Sf1-EGFP* and *Sry-EGFP/Sox9-ECFP* data sets. (A) To cross-validate the analysis, we determined whether genes primed in the *Sf1-EGFP* data (removing interstitial/stromal genes, Figure 6E) were also identified as primed in the *Sry-EGFP/Sox9-ECFP* data (all genes with a priming pattern, Figure 5B). Some of the same primed genes were identified in both data sets (blue). Many more of the *Sf1* primed genes were already sexually dimorphic in the *Sry-EGFP/Sox9-ECFP* cells by E11.5 (light and dark green). This is not surprising since primed genes were already becoming dimorphic at E11.5 based on the *Sf1-EGFP* data (Figure S5G). Some of the genes that were already sexually dimorphic in the E11.5 *Sry-EGFP/Sox9-ECFP* cells showed some indication of previous priming in the *Sry-EGFP/Sox9-ECFP* data (light green). These genes met the same requirements for defining a primed pattern outlined in the Materials and Methods, but rather than being identical at E11.5, the sex for which the gene was primed had higher expression at E11.5. This pattern is illustrated in (B) by the graphs of the log-transformed, normalized intensity values for the *Sf1-EGFP* (black) and *Sry-EGFP/Sox9-ECFP* (blue) cells for the gene *Dock4*. The error bars are standard error. Together, these primed or E11.5 dimorphic categories account for over half of the *Sf1-EGFP* primed genes, indicating both arrays showed consistent results for many genes. A number of *Sf1-EGFP* primed genes (48%) were not identified as primed or sexually dimorphic in *Sry-EGFP/Sox9-ECFP* cells at E11.5 (yellow). This was expected as probe sets for the two arrays, as well as the cell types collected, were different. The identification of similar patterns for the same genes in these two different data sets despite their differences gives us confidence in the results. The gene lists for these analyses are provided in Dataset S5F.
(TIF)
