# Supplementary figures and images for: Temporal Transcriptional Profiling of Somatic and Germ Cells Reveals Biased Lineage Priming of Sexual Fate in the Fetal Mouse Gonad
Source: PLoS Genet. 2012 Mar 15;8(3):e1002575. doi: 10.1371/journal.pgen.1002575 (PMC3305395; doi:10.1371/journal.pgen.1002575)

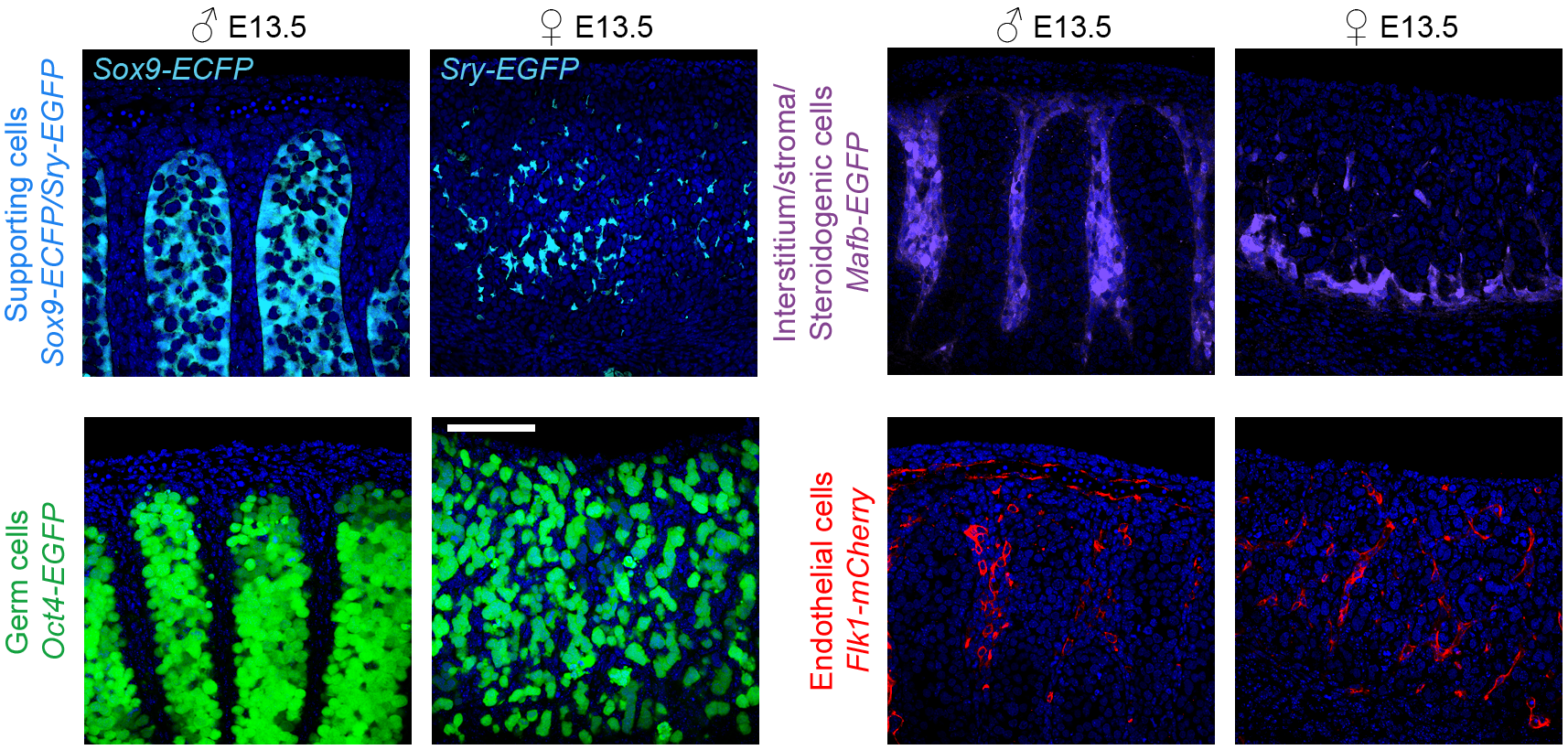

Supplement: Figure S1 — Lineage-specific fluorescent tags used for FACS. Images of E13.5 XY and XX gonads with DAPI (blue) and each fluorescent marker used: Sox9-ECFP and Sry-EGFP (cyan) labeling supporting cells, Mafb-EGFP (purple) labeling the interstitial/stromal cells, Oct4-EGFP (green) labeling germ cells, and Flk1-mCherry (red) labeling endothelial cells. Scale bar = 100 µm. (TIF) [file pgen.1002575.s006.tif]

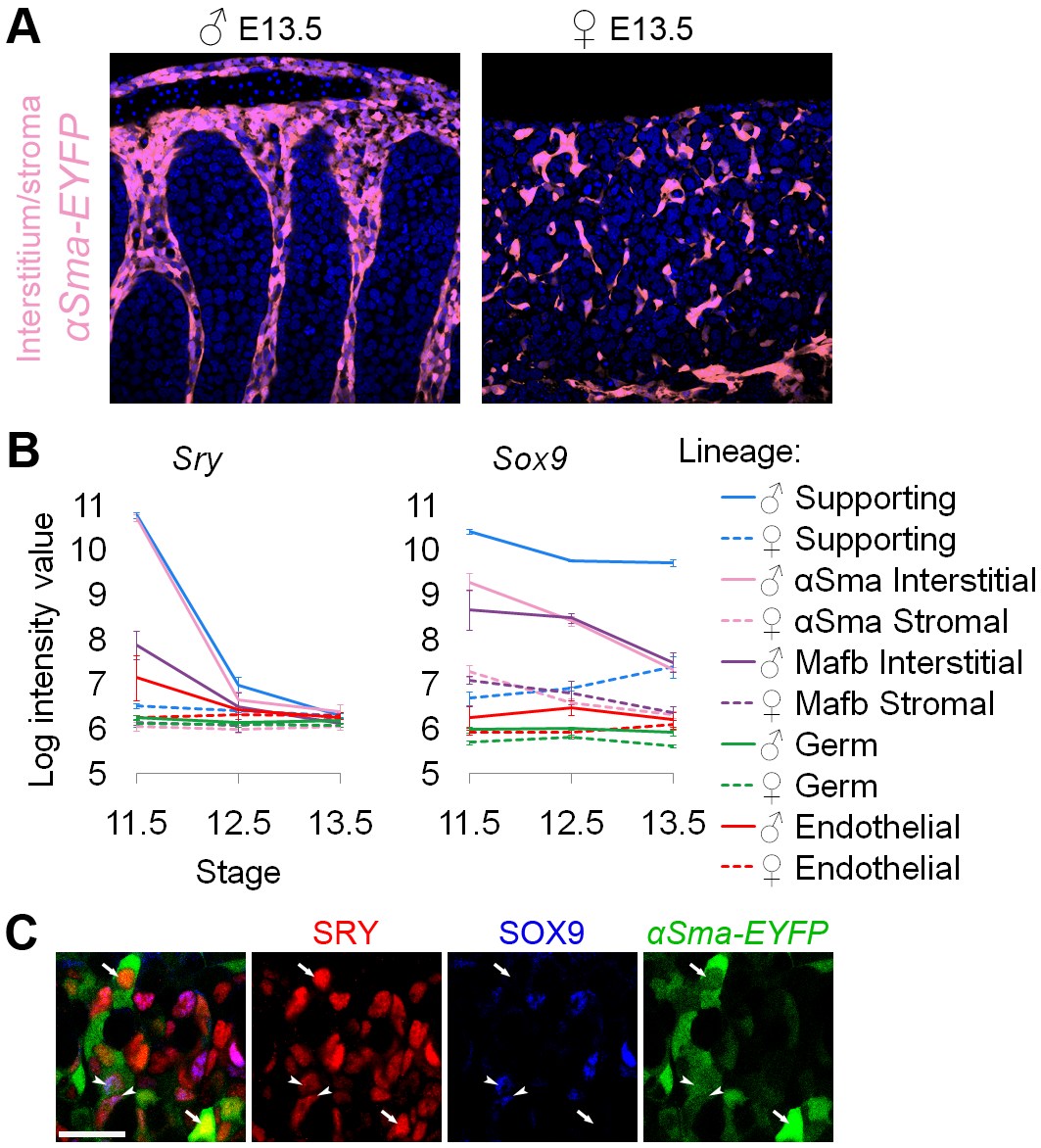

Supplement: Figure S2 — αSma-EYFP labeled a heterogeneous population containing supporting cell precursors. (A) Images of E13.5 XY and XX gonads with DAPI (blue) and αSma-EYFP (pink) labeling the interstitial/stromal cells. (B) Graphs of the log-transformed, normalized intensity values. The error bars are standard error. The Sry transcript is expressed at similarly high levels in both XY supporting cells and αSma-EYFP cells at E11.5, and declines rapidly in both cell types. Expression of Sry is lower in the Mafb-EGFP cells. However, the pattern seen with Sry did not hold true for most supporting cell markers: Sox9 is expressed at a lower level in both αSma-EYFP and Mafb-EGFP cells than in supporting cells. (C) SRY and SOX9 proteins are also present in αSma-EYFP cells. Antibodies against SRY (red) and SOX9 (blue) co-label αSma-EYFP (green) cells. Cells with αSma-EYFP and SRY alone are indicated with arrows, whereas cells with αSma-EYFP, SRY, and SOX9 are indicated with arrowheads. Scale bar = 25 µm. This suggests that αSma-EYFP is expressed in a heterogeneous population of early gonadal cells containing supporting cell progenitors. (TIF) [file pgen.1002575.s007.tif]

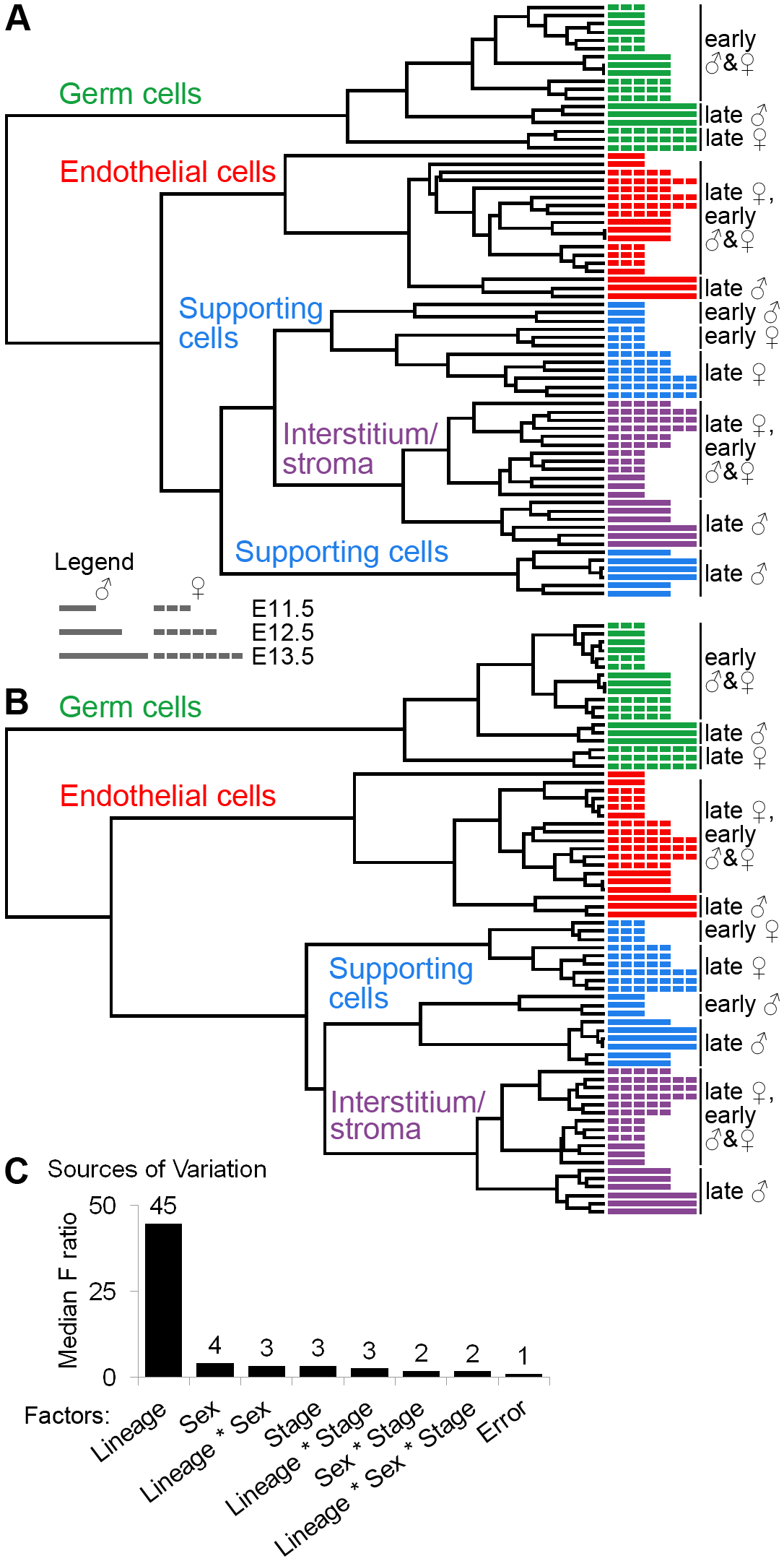

Supplement: Figure S3 — Alternative methods showed generally similar patterns indicating the importance of lineage, sex, and stage. Clustering dendrograms of the individual arrays generated using (A) Average linkage with Euclidean distance as a distance metric and (B) Complete linkage with Pearson's dissimilarity as a distance metric. Consistent with Figure 2A, the arrays cluster primarily by lineage, and secondarily by sex and stage. The largest differences were in the relationship of the somatic populations to each other, although the same clusters could always be identified. (C) Examining the sources of variation with the median F ratio shows a similar pattern to the mean F ratio (Figure 2B) with the primary source of variation being lineage. (TIF) [file pgen.1002575.s008.tif]

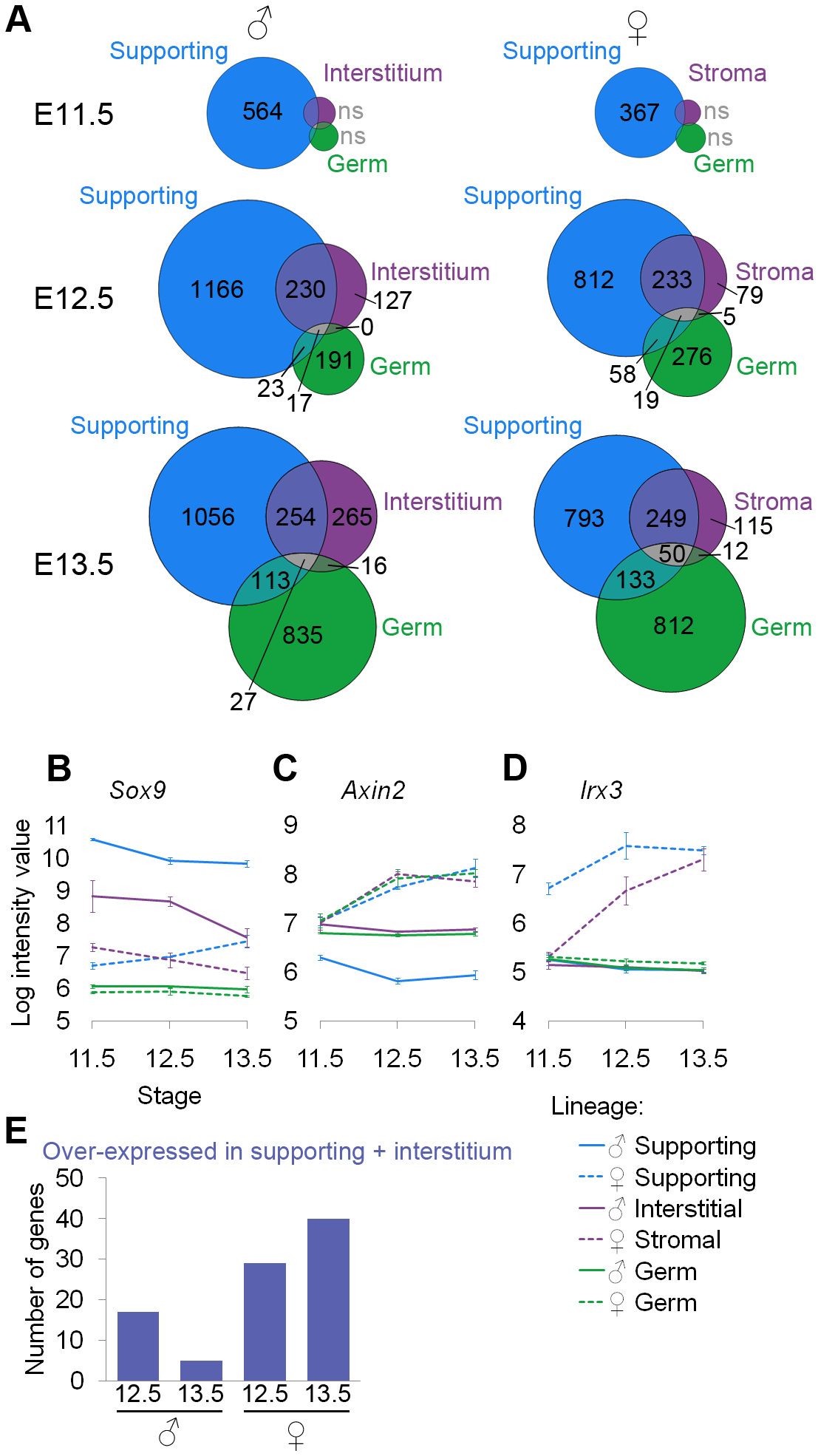

Supplement: Figure S4 — Overlap of genes that are sexually dimorphic in at least one lineage. (A) The number of genes over-expressed (“sexually dimorphic”) in XY or XX cells of each lineage at each stage. Genes were identified by a single pairwise comparison between XX and XY cells for each lineage at each stage (Dataset S3A). Many more genes are identified here than in Figure 3A because additional pairwise comparisons were performed to restrict the analysis to genes that showed lineage-specificity in Figure 3A. The area proportional Venn diagrams were generated using Venn Diagram Plotter v1.4.3740 from PNNL and OMICS.PNL.GOV (http://omics.pnl.gov/software/VennDiagramPlotter.php). The sizing of the Venn diagrams relative to each other is approximate. Endothelial cells were not analyzed. The numbers shown indicate the number of genes exclusively in each portion of the diagram (except for E11.5 for which the total number of genes dimorphic in the supporting cells is shown). Lists marked “ns” had a false positive rate >20% (Dataset S3B). The overlapping areas on the Venn diagrams indicate genes sexually dimorphic in multiple lineages. Most genes dimorphic in the XX stroma were also dimorphic in another lineage. (B–D) Graphs of the log-transformed, normalized intensity values. The error bars are standard error. Endothelial cell values are not shown. (B) Many genes sexually dimorphic in multiple lineages were over-expressed in one of the lineages, as was the case for Sox9. However, this could be explained by the low and variable contamination expected after FACS. To address this issue, we used antibody stains of sorted cells to estimate that the XY E13.5 germ cells had <1% contamination with supporting cells, but the XY E13.5 interstitium was more variable and had between 1% and 15% supporting cell contamination (data not shown). Therefore, patterns similar to Sox9 were not further analyzed. (C–D) However, not all genes sexually dimorphic in multiple lineages had a pattern consistent with low l [file pgen.1002575.s009.tif]

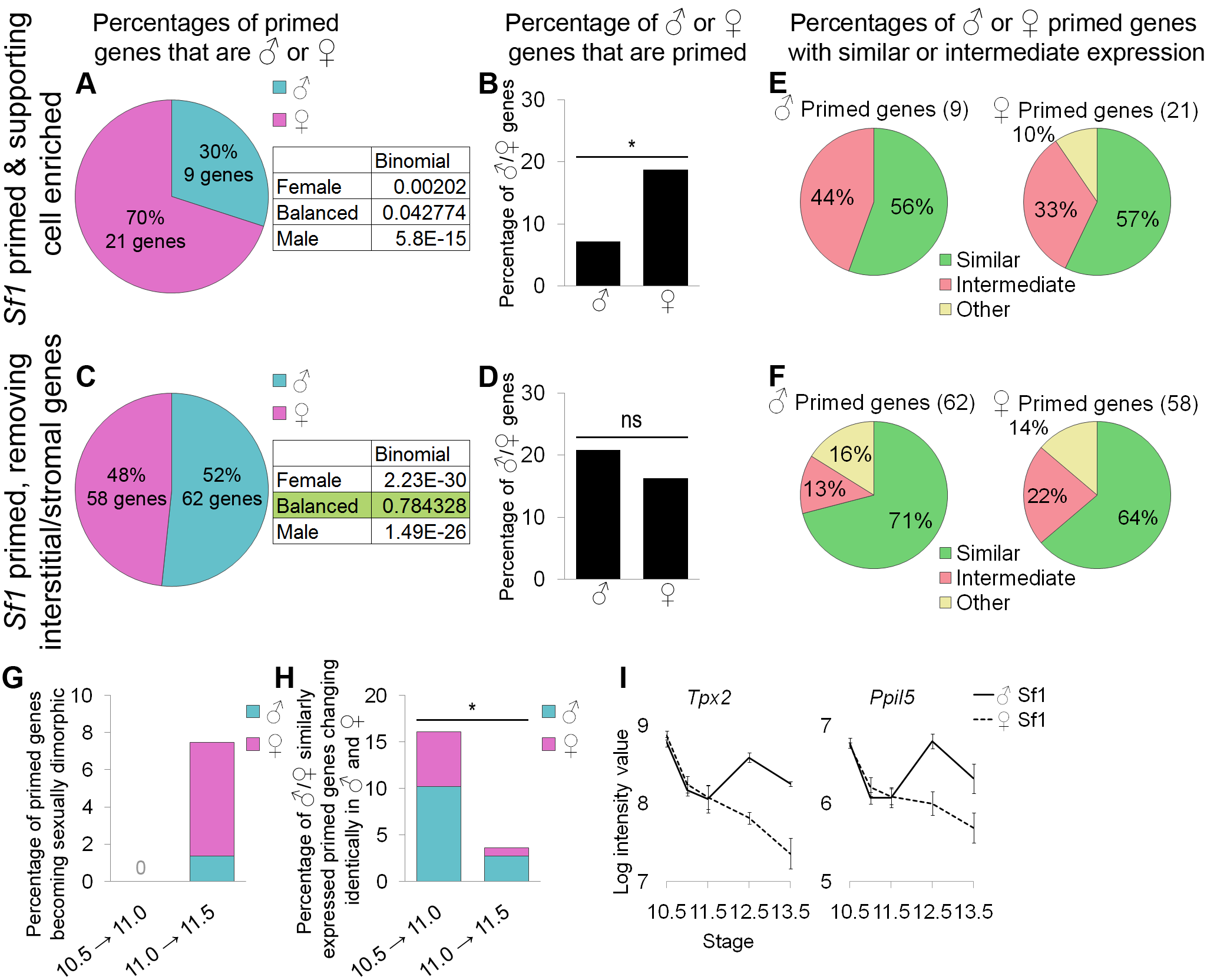

Supplement: Figure S5 — E10.5 Sf1-EGFP primed genes generally supported female-biased priming, but the E11.0 analysis was more informative. The analysis of the Sf1-EGFP primed genes (beginning the analysis at E10.5 and comparing to Sf1-EGFP cells at E12.5) was also limited to genes enriched in the Sry-EGFP/Sox9-ECFP supporting cells at E12.5 (A, B, and E) or to those genes identified by removing interstitial/stromal genes (C, D, and F). (A and C) The percentages of primed genes that were male-primed and female-primed. The first, but not the second, method showed a female bias. The boxes contain the p-values from the binomial test with the expected percentages of the extreme models. The balanced model can be rejected with the first (A), but not the second (C), method. (B and D) Examining the percentage of male or female genes that were primed similarly showed a significant (*) bias toward the female pathway, as determined by the hypergeometric test (p-value<0.05), for the first (B), but not the second (D), method (ns). (E and F) The primed genes for both sexes are predominantly similarly expressed in progenitors and E12.5 differentiated cells. However, E10.5 may not be the appropriate starting point for the priming analysis. (G) No primed genes (identified by removing interstitial/stromal genes) became dimorphic between E10.5 and E11.0 (“0”). Thus, starting the analysis at E11.0 does not result in the loss of any information. Starting at E11.0 is also preferable because it is closer to the divergence point. (H) Between E10.5 and E11.0, 98% of the primed genes were identically expressed in XX and XY samples at both E10.5 and E11.0, and 76% of the primed genes were identically expressed in XX and XY samples at both E11.0 and E11.5 (data not shown). Of these genes, 16% were changing expression level in the same way (see I) in both XX and XY cells between E10.5 and E11.0, whereas only 4% fell in this category between E11.0 and E11.5. This difference was significant (*), as determined by the hyp [file pgen.1002575.s010.tif]

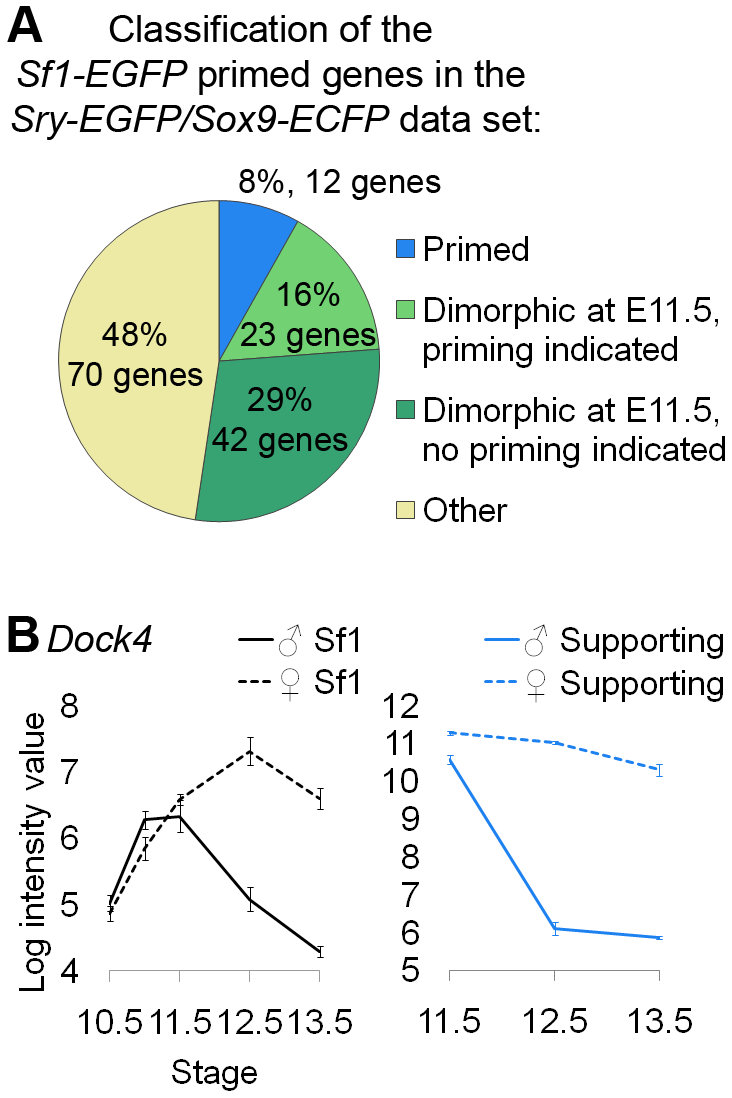

Supplement: Figure S6 — Overlap of primed genes between the Sf1-EGFP and Sry-EGFP/Sox9-ECFP data sets. (A) To cross-validate the analysis, we determined whether genes primed in the Sf1-EGFP data (removing interstitial/stromal genes, Figure 6E) were also identified as primed in the Sry-EGFP/Sox9-ECFP data (all genes with a priming pattern, Figure 5B). Some of the same primed genes were identified in both data sets (blue). Many more of the Sf1 primed genes were already sexually dimorphic in the Sry-EGFP/Sox9-ECFP cells by E11.5 (light and dark green). This is not surprising since primed genes were already becoming dimorphic at E11.5 based on the Sf1-EGFP data (Figure S5G). Some of the genes that were already sexually dimorphic in the E11.5 Sry-EGFP/Sox9-ECFP cells showed some indication of previous priming in the Sry-EGFP/Sox9-ECFP data (light green). These genes met the same requirements for defining a primed pattern outlined in the Materials and Methods, but rather than being identical at E11.5, the sex for which the gene was primed had higher expression at E11.5. This pattern is illustrated in (B) by the graphs of the log-transformed, normalized intensity values for the Sf1-EGFP (black) and Sry-EGFP/Sox9-ECFP (blue) cells for the gene Dock4. The error bars are standard error. Together, these primed or E11.5 dimorphic categories account for over half of the Sf1-EGFP primed genes, indicating both arrays showed consistent results for many genes. A number of Sf1-EGFP primed genes (48%) were not identified as primed or sexually dimorphic in Sry-EGFP/Sox9-ECFP cells at E11.5 (yellow). This was expected as probe sets for the two arrays, as well as the cell types collected, were different. The identification of similar patterns for the same genes in these two different data sets despite their differences gives us confidence in the results. The gene lists for these analyses are provided in Dataset S5F. (TIF) [file pgen.1002575.s011.tif]
